# Insights from animal models: Dissecting the independent roles of oxygen and nutrients in the fetal origins of cardiovascular disease

**DOI:** 10.1113/JP289670

**Published:** 2026-03-11

**Authors:** Melanie R. Bertossa, Jack R. T. Darby, Ashley S. Meakin, Mitchell C. Lock, Steven K. S. Cho, Christopher K. Macgowan, Mike. Seed, Michael D. Wiese, Janna L. Morrison

**Affiliations:** ^1^ Early Origins of Adult Health Research Group, Robinson Research Institute, School of Pharmacy and Biomedical Sciences, College of Health Adelaide University Adelaide Australia; ^2^ Department of Physiology, Faculty of Medicine University of Toronto Toronto Canada; ^3^ Translational Medicine Hospital for Sick Children Toronto Canada; ^4^ Department of Medical Biophysics, Faculty of Medicine University of Toronto Toronto Canada; ^5^ Division of Cardiology Hospital for Sick Children Toronto Canada; ^6^ Department of Paediatrics, Faculty of Medicine University of Toronto Toronto Canada

**Keywords:** cardiovascular disease, growth restriction, heart, fetal, nutrients, oxygen

## Abstract

Depending on the pregnancy complication, substrate delivery to a developing fetus can be reduced, changing the course of fetal growth below the genetically determined *in utero* growth potential, resulting in fetal growth restriction (FGR). FGR is linked to an increased risk of developing cardiovascular disease (CVD) in later life. When caused by placental insufficiency, FGR is characterized by fetal hypoxaemia and hypoglycaemia due to reduced substrate supply to the fetus. However, other common pregnancy complications exist, where fetal hypoxaemia or hypoglycaemia may or may not occur. It is therefore necessary to understand the independent and synergistic contributions of hypoxaemia and hypoglycaemia to the fetal origins of CVD. In doing so, this knowledge will aid in the development of intervention strategies. The aim of this review is to provide mechanistic insights by comparing findings across different paradigms of developmental programming using animal models of FGR, with consideration of the timing, duration, and severity of the insult, and the ventricles and fetal sex studied.

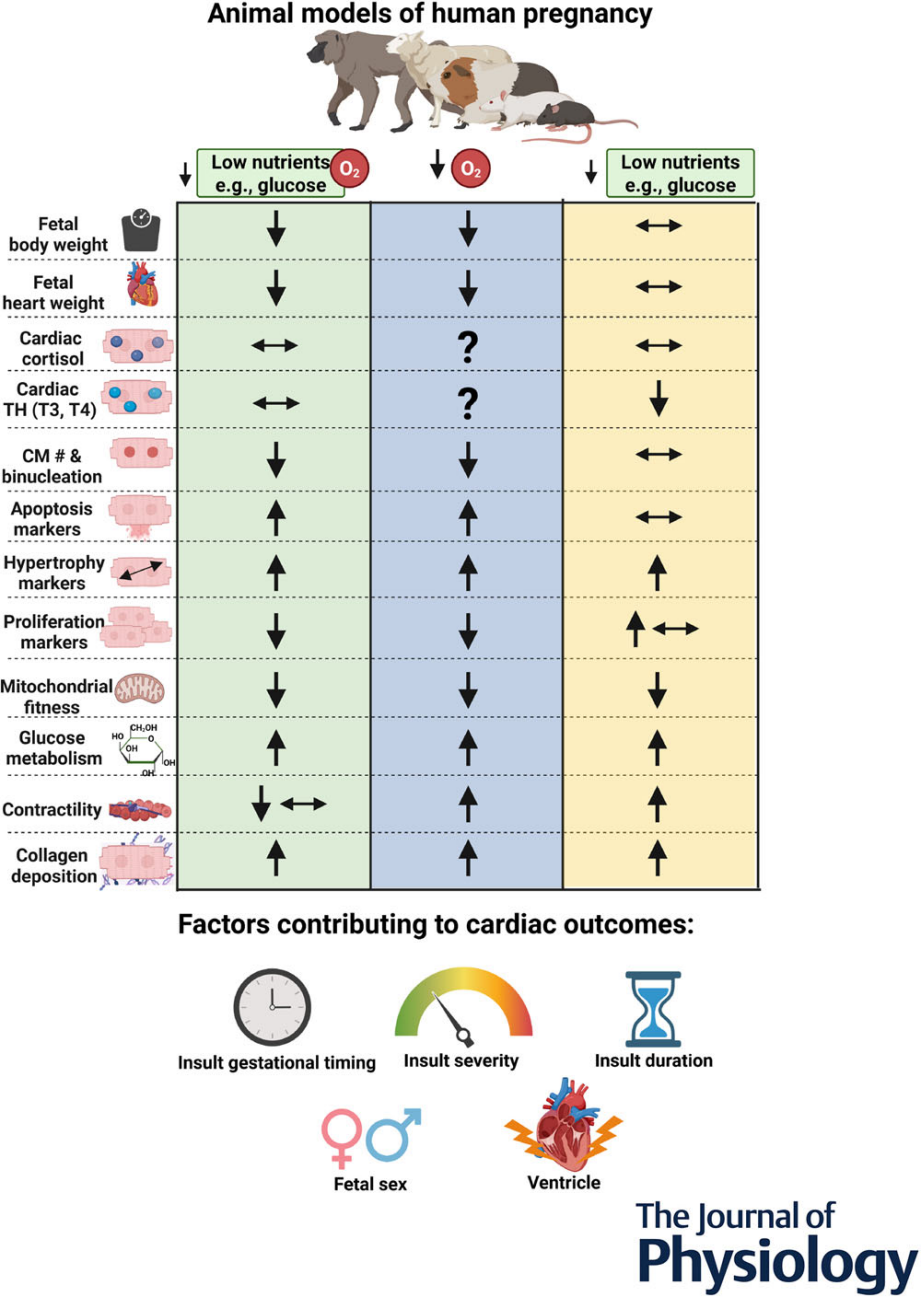

## Graphical abstract

**Abstract figure legend** This review utilized animal models of complicated human pregnancies that result in reduced fetal nutrient or oxygen delivery, or combined nutrient and oxygen delivery, to elucidate their independent and/or synergistic contributions to the development of high‐risk cardiac phenotypes. Across models, common cardiac phenotypic endpoints emerge regardless of the specific substrate deficiency; however, the upstream mechanisms driving these outcomes can differ. The review also focused on how these mechanisms are influenced by gestational timing, the duration and severity of the substrate deficiency, fetal sex and ventricular specificity. Question marks indicate gaps in the existing literature. ↔, no change; ↑, increase; ↓, decrease; CM, cardiomyocyte; TH, thyroid hormones; T3; triiodothyronine; T4, thyroxine.

## Introduction

Normal fetal development requires adequate substrate (oxygen (O_2_) and nutrients) transfer from the maternal to fetal circulation. Disruption in substrate transfer can alter the trajectory of fetal growth, leading to babies being born with pathological fetal growth restriction (FGR) (Economides & Nicolaides, 1989; Economides et al., 1991; Nicolaides et al., 1989; Owens, 1995; Robinson et al., 1994; Soothill et al., 1987). Importantly, FGR is not synonymous with small for gestational age (SGA; <10th birthweight centile), where babies are genetically destined to be small but are otherwise healthy. In FGR, genetic growth potential is not met. Indeed, human epidemiological studies highlight an association between FGR and an increased risk of cardiovascular disease (CVD) in later life (Barker, 2000; Barker et al., 2010). This discovery underpins the developmental origins of health and disease hypothesis, which states that deficiencies in the intrauterine environment result in permanent alterations in key tissue and organ systems during fetal life that persist after birth, manifesting as chronic non‐communicable diseases such as metabolic syndrome and CVD (Barker, 1990, 2000; McMillen & Robinson, 2005).

**Figure 1:**
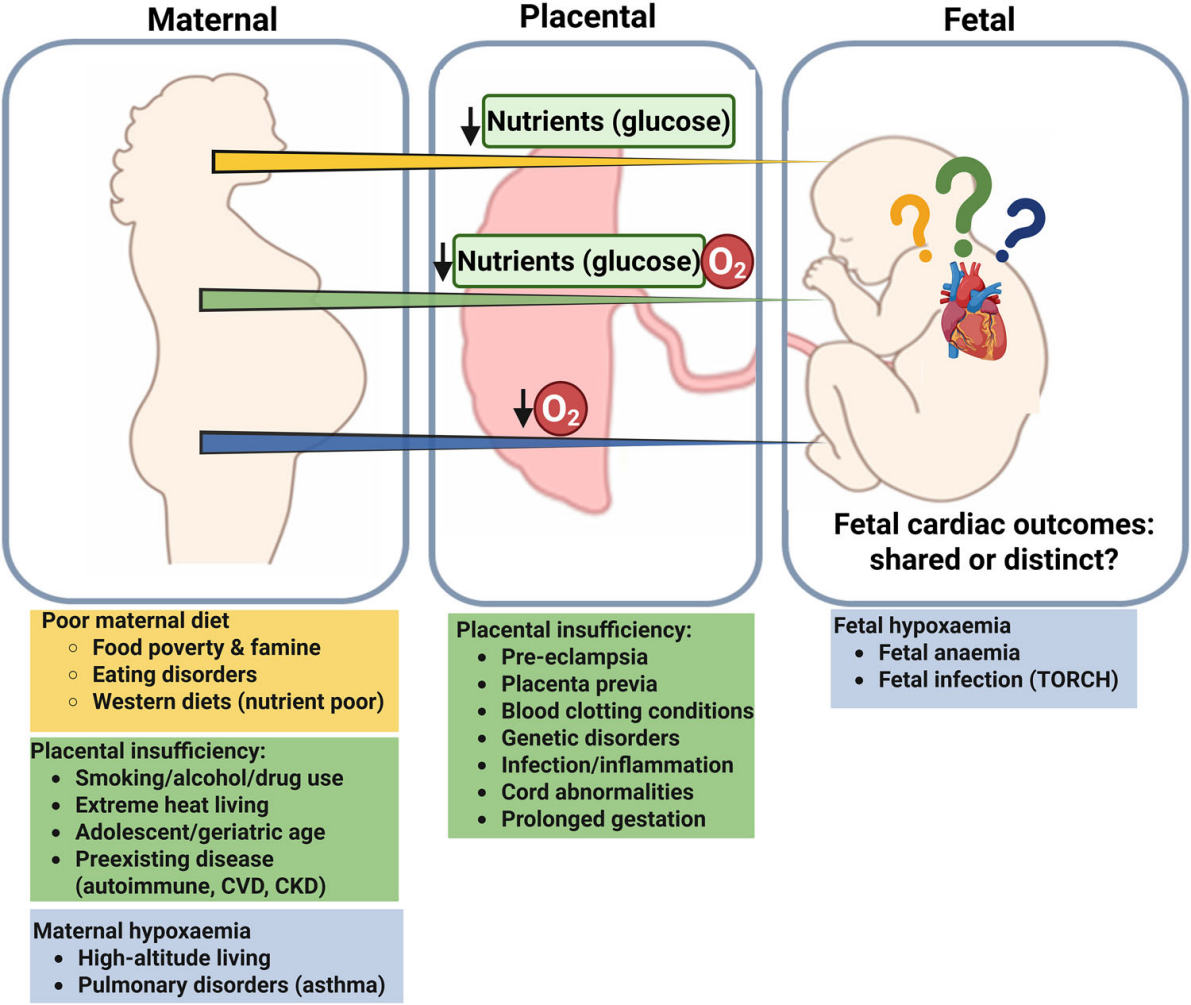
Pregnancy complications leading to fetal hypoxaemia and/or hypoglycaemia and their impact on the developing fetal heart remain largely unknown During pregnancy, oxygen (O_2_) and nutrients, such as glucose, are transferred across the placenta from the maternal to the fetal unit. Many pregnancy complications of maternal, placental or fetal origin can lead to reduced oxygen in the fetal circulation (blue), reduced nutrients such as glucose (yellow) or reduced oxygen and glucose (green) to the developing fetus. However, the independent and potentially synergistic roles of oxygen and glucose on fetal heart development due to poor maternal diet (yellow), placental insufficiency (green) and hypoxaemia (blue) remain unclear. Cardiovascular disease (CVD), chronic kidney disease (CKD); TORCH (toxoplasmosis, other infections (syphilis, varicella and parvovirus), rubella, cytomegalovirus and herpes simplex).

Elucidating the underlying mechanisms linking FGR to CVD is key to developing targets for intervention that may be employed to protect against the fetal origins of CVD. This has proven to be a challenge, as the transfer of many substrates across the maternal–placental–fetal unit can be restricted in complicated pregnancies. For instance, FGR can be due to factors of maternal, placental or fetal origin that reduce the capacity of the placenta to deliver O_2_ and nutrients (primarily glucose, the major fuel source in fetal life) but also other nutrients such as amino acids and fatty acids (FAs), termed placental insufficiency (PI), of which fetal hypoxaemia and hypoglycaemia are hallmarks (Economides & Nicolaides, 1989; Economides et al., 1991; Nicolaides et al., 1989; Soothill et al., 1987). However, causes of FGR are heterogeneous and not solely due to PI, such that other pregnancy complications may be associated with either fetal hypoxaemia or hypoglycaemia. For example, maternal nutrient restriction (MNR) reduces fetal nutrient delivery, resulting in fetal hypoglycaemia, which, depending on the timing, severity and duration of the insult, can leave an intact uteroplacental unit that maintains fetal O_2_ delivery (Morrison, 2008) (Fig. 1). While PI may be a leading cause of FGR, MNR is also associated with elevated CVD risk (Roseboom et al., 2001; Thornburg & Valent, 2024), a contribution to CVD burden that should not be overlooked, as the worldwide prevalence of undernutrition in adolescent girls and women is high, with approximately one billion recorded in 2023 (Xu et al., 2024). This can be due to a range of factors, including food insecurity among women living in low‐ and middle‐income countries, cultural beliefs and morning sickness (Olajide et al., 2024). In higher‐income countries, poverty or body‐image concerns can drive restrictive eating habits and/or the continuation of weight loss medication into pregnancy despite the recommendation to stop (Crozier et al., 2009; Nuako et al., 2023).

The broader molecular mechanisms underpinning programmed CVD risk in FGR have recently been reviewed (Dimasi et al., 2023; Wu et al., 2025). However, the current review aims to tease apart the independent and synergistic contributions of the two major substrates used by the heart in fetal life, oxygen and glucose, leading to poor cardiac molecular signatures. In addition to substrate specificity, the timing, severity, and duration of altered substrate delivery (Darby et al., 2020; Morrison, 2008), as well as ventricle and fetal sex, are considered.

### Animal models that have similar placental and fetal heart development to humans

In complicated human pregnancies, the molecular mechanisms that regulate the development of the fetal heart cannot be measured directly, and assessing long‐term outcomes requires lengthy longitudinal and logistically challenging follow‐up studies spanning the life course (Oken et al., 2023). Animal models can be used to recapitulate human pregnancy, including non‐human primates (such as macaques and baboons), sheep and rodents (including guinea pigs, mice and rats), each with reproductive similarities and differences to humans, which must be carefully considered when translating relevant findings into the human context. Like humans, baboons and sheep have longer gestation lengths and produce predominantly singletons or twins (Cox et al., 2017; Morrison, Berry, Botting, et al., 2018). While guinea pigs can have litter sizes of 2–4, the litter sizes of rats and mice are larger (6–12), with shorter gestation lengths. However, the shorter gestation and lifespan of rats and mice are beneficial for studies of long‐term follow‐up or intergenerational impacts. More important than the length of gestation is the timing of heart maturation compared with humans.

**Figure 2:**
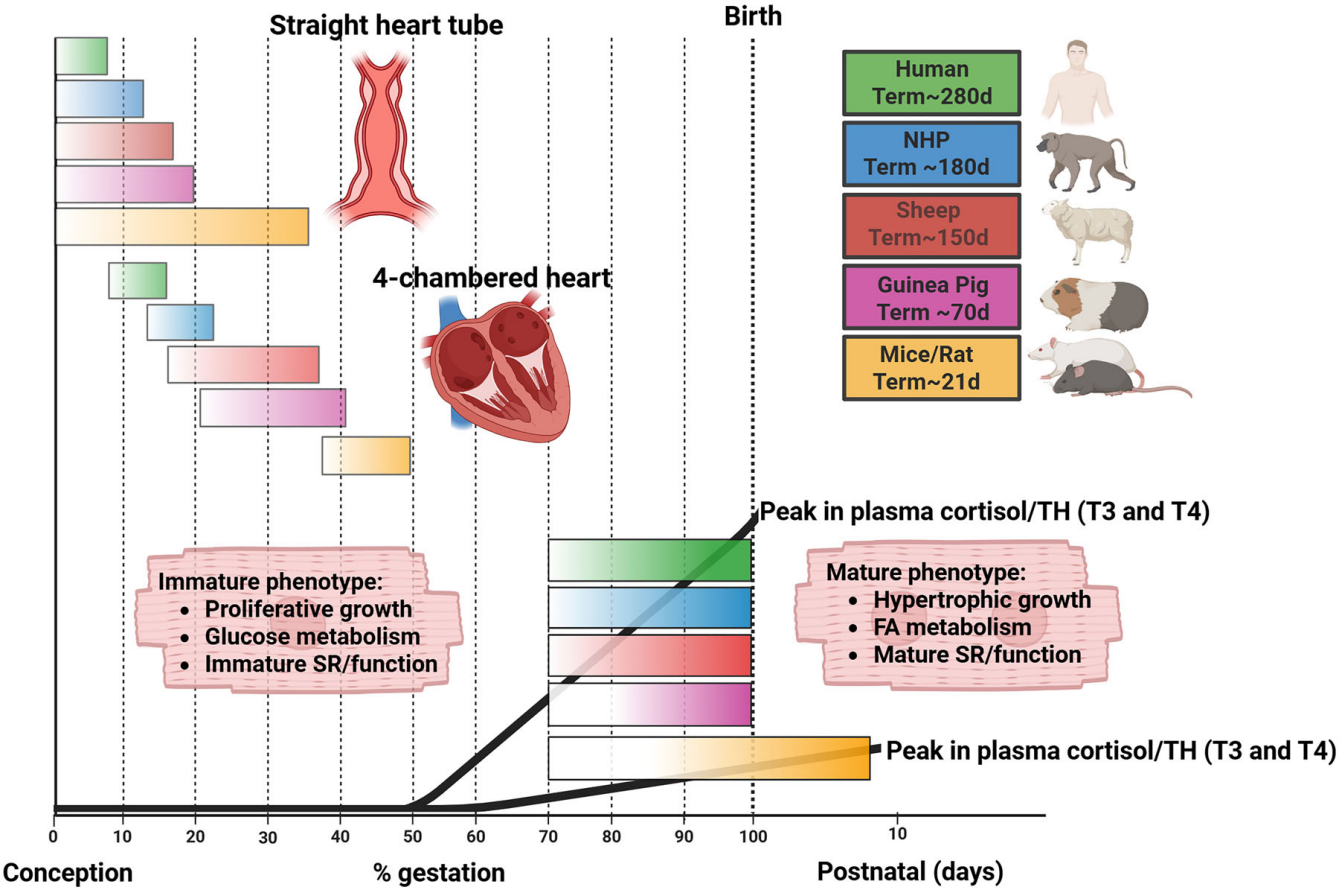
Comparative timing of cardiogenesis in early gestation and cardiomyocyte (CM) maturation in late gestation across different species available for use in cardiovascular research During embryonic development, the primitive heart tube emerges at 22 days gestational age (dGA) in humans (∼8% gestation), 24 dGA in non‐human primates (NHP; ∼13% gestation), 25 dGA in sheep (∼16% gestation), 14–15 dGA in guinea pigs (20% gestation) and 7.5 dGA in mice/rats (∼35% gestation). This is followed by the looping and twisting of the straight heart tube that eventually leads to the four‐chambered heart by 49 dGA in humans, 41 dGA in NHPs, 56 dGA in sheep, 29 dGA in guinea pigs and 12 dGA in mice and rats. Ongoing remodelling of CM growth, metabolism and contractility continues, coinciding with the timing of the prepartum surge in plasma cortisol concentration that leads to increased thyroid hormone (TH) concentration, with most species reaching mature phenotypes before birth (humans, baboons, sheep, guinea pigs) or after birth (rats/mice).

Early pregnancy marks a period of rapid cardiogenesis, beginning with the formation of the primitive heart tube. Looping creates the framework for the mature four‐chambered heart, achieved via further twisting and septation. Non‐human primates are closely related to humans, with the most conserved genetic makeup and timing of heart development (Cox et al., 2017; Nakamura et al., 2021). Sheep and guinea pigs have a mature heart structure by the end of the first trimester, whereas in rats and mice this does not occur until mid‐gestation (Fig. 2) (Fitzsimons et al., 2022; Valasi et al., 2017). In humans, primates, sheep and guinea pigs, cardiomyocytes (CMs) transition to a mature phenotype by birth, initiated by a surge in cortisol and thyroid hormones (THs; Fig. 2) (Fowden et al., 1998; Thornburg et al., 2011). This transition is characterized by an upregulation of the molecular machinery required to utilize free FAs for energy, CM growth primarily via hypertrophy, either CM multinucleation/endoreplication or entry into a quiescent, non‐proliferative state and phenotypic switches in contractile properties. These are in parallel with a transition away from primarily utilizing glucose as a metabolic fuel, hyperplastic CM growth, and functional immaturity (Amanollahi et al., 2025; Dimasi et al., 2023; Jonker et al., 2010; Morrison et al., 2018). In comparison, rats and mice are born with the immature cardiac profile and phenotypes associated with maturity are not reached until shortly after the first neonatal week (Li et al., 1996) (Fig. 2). Although this delay in the developmental window in rats can be exploited as a time when intervention could enhance proliferation and normalize CM endowment (Black et al., 2012). In contrast, for humans, baboons, sheep and guinea pigs, late gestation is a vulnerable period in heart maturation where changes in oxygen and glucose delivery, the main substrates in fetal life, can impact CM development (Amanollahi et al., 2025; Jonker, Louey, Giraud, et al., 2015; Rolph et al., 1982). While animal models are useful, reproductive variability between species can introduce challenges when comparing study outcomes and translating findings into the human context.

The placenta must also be considered when selecting animal models of human pregnancy, as it sits at the interface of substrate transfer from mother to fetus. Placental anatomy is most similar between non‐human primates and humans (Matsumoto et al., 2023), with guinea pigs also sharing many morphological similarities (Morrison et al., 2018), followed by haemochorial rat placentas (Hemberger et al., 2020). While this contrasts with the cotyledonary placenta of sheep, the fetal oxygen consumption rate is similar between sheep and humans (Saini et al., 2020).

**Figure 3:**
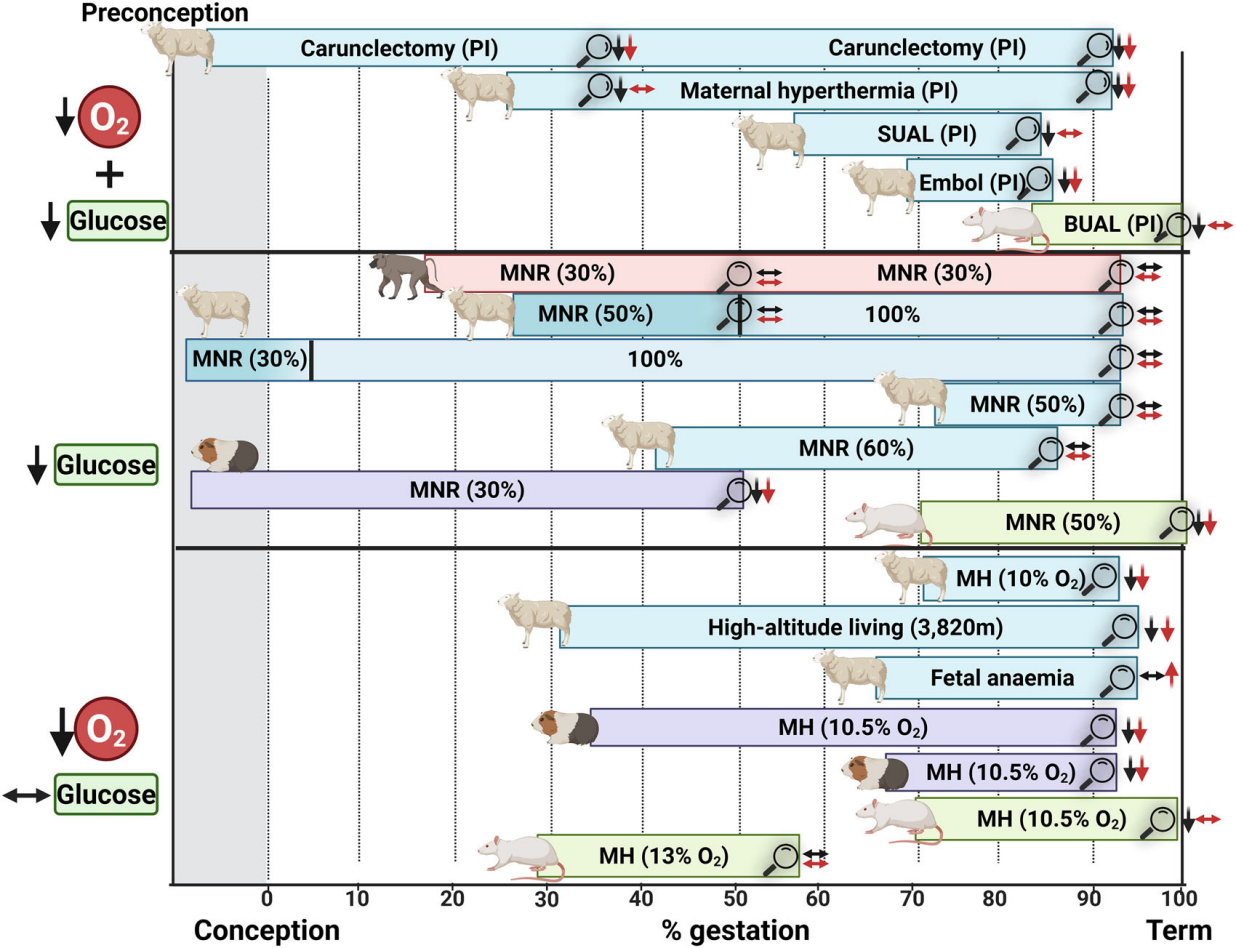
There are a range of animal models of placental insufficiency (PI; reduced oxygen and glucose), maternal nutrient restriction (MNR; reduced glucose) and fetal hypoxaemia (maternal hypoxia (MH), high‐altitude (HA) living and fetal anaemia) that can be initiated in early or late gestation with differing degrees of severity and duration that report cardiac outcomes Magnifying glass indicates gestational ages at which fetal heart development has been investigated. Black arrows indicate direction of change for fetal body weight, red arrows indicate direction of change for total fetal heart weight. Data on body and heart outcomes were obtained for PI models (Barry et al., 2006; Brown et al., 2012; Bubb et al., 2007; Chou & Chen, 2023; Morrison et al., 2007; Polglase et al., 2016; Regnault et al., 2007; Zhang et al., 2024), MNR models (Darby et al., 2018b; Dong et al., 2008; Edwards et al., 2001; Han et al., 2004; Lemley et al., 2012; Lesage et al., 2001; Lie et al., 2013; Masoumy et al., 2018; Pereira et al., 2021; Pereira et al., 2023; Vonnahme et al., 2003; Zouridis et al., 2021) and fetal hypoxaemia models (Brain et al., 2015; Davis et al., 2018; Giussani et al., 2012; Jonker et al., 2010, 2011; Kamitomo et al., 1992; Song et al., 2021; Tong et al., 2011; Tong et al., 2022; Williams et al., 2005). BUAL, bilateral umbilical artery ligation; Embol, embolization; SUAL, single umbilical artery ligation.

This review will therefore focus primarily on outcomes in mammals in which the heart matures before birth, like humans. However, mechanistic insights will be drawn from other species where appropriate. As such, a variety of animal models exist to recapitulate human PI (Fig. 3) (Morrison, 2008), reducing uteroplacental blood flow and placental weight at term (Bell et al., 1987; Regnault et al., 2007), resulting in fetal hypoxaemia and hypoglycaemia (Miller et al., 2009; Poudel et al., 2015). A range of animal models have also been developed to recapitulate early‐ and late‐onset global restriction of maternal nutrients, which can result in reduced delivery of nutrients to the fetus, of which fetal hypoglycaemia is best characterized. If onset is in late gestation, placental weight is maintained and there is no fetal hypoxaemia; however, early‐onset models can impair placental development, leading to reduced placental and fetal weight, like PI (Fig. 3) (Cho et al., 2026; Darby et al., 2018a; Steinhauser et al., 2021; Vonnahme et al., 2003). Models used to induce fetal hypoxaemia in sheep and rodents include recapitulating the natural hypobaric hypoxia experienced by women living at high altitude (∼3820 m above sea level) (Giussani, 2021), fetal anaemia induced by isovolumic haemorrhage (Jonker et al., 2010), or maternal hypoxia (MH) via hypoxia chambers in sheep, guinea pigs, rats and mice (Fig. 3) that allow for controlled reductions in maternal inspired oxygen (Botting et al., 2018; Brain et al., 2015; Tong et al., 2022).

### Placental insufficiency: the impact of low oxygen and glucose on fetal heart development

In models of PI, fetal body weight is reduced (∼30% on average across models; Fig. 3) (Drake et al., 2022; Morrison et al., 2007; Polglase et al., 2016; Rock et al., 2026; Thompson et al., 2011). Early pregnancy is a vulnerable period for the rapidly developing fetal heart, but so is late gestation when CMs are undergoing maturation, such that early‐ and late‐onset PI can similarly reduce fetal heart weight (Fig. 3) (Bubb et al., 2007; Chang et al., 2024; Jonker, Kamna, LoTurco, et al., 2018; Murotsuki et al., 1997; Wang et al., 2011). In some instances, reduced heart weight can remain into adulthood (Black et al., 2012; Louey et al., 2000; Paz et al., 2019; Vranas et al., 2017). Reduced fetal heart weight may be partly attributed to a reduction in total CM number in late gestation (Black et al., 2012; Botting et al., 2014; Jonker, Kamna, LoTurco, et al., 2018; Mattern et al., 2023; Morrison et al., 2007) that persists through adolescence into adulthood (Black et al., 2012; Botting et al., 2018; Stacy et al., 2009; Vranas et al., 2017).

A common finding among many models of PI with FGR is a reduction in insulin‐like growth factor (IGF) 1 in the fetal circulation (Brown et al., 2022; Dong & Thompson, 2006; Eremia et al., 2007; Jones et al., 1990; Owens et al., 1994; Wali et al., 2012; Woodall et al., 1996a), which is correlated with reduced CM numbers (Jonker, et al., 2018). Indeed, IGF1 is a key regulator of the CM population (Thornburg et al., 2011). Despite this, there is limited evidence for a change in cardiac IGF1 mRNA expression by PI; however, IGF1 receptor (IGF1R) mRNA expression is reduced, indicating reduced downstream hyperplastic growth signalling (Kind et al., 1995; Wang et al., 2011; Zhang et al., 2024). Despite this, there is no change in the number of CMs undergoing proliferation in late gestation (e.g. CMs positive for Ki‐67). Instead, increased cardiac apoptosis around the time of PI onset contributes to the reduced CM endowment in early‐ and late‐gestation fetal sheep, guinea pig and rat hearts (Black et al., 2012; Chou & Chen, 2023; Guo et al., 2025; Mattern et al., 2023; Zhang et al., 2024). In instances where apoptosis is absent despite PI, gene signatures indicative of CMs primed for apoptosis but not fully committed are evident in late gestation (Botting et al., 2014; Simões et al., 2018). By late gestation, the capacity to replace CMs is limited as the ability of CMs to proliferate declines, and there is no compensatory increase in markers of cell cycle progression (Barooni et al., 2026; Kind et al., 1995; Louey et al., 2007; Morrison et al., 2007; Zhang et al., 2024). Instead, the remaining CMs compensate by hypertrophy, with the size of CMs and markers involved in the pathological hypertrophic pathways (IGF2R) increased in the early‐ and late‐gestation fetal heart (Murotsuki et al., 1997; Wang et al., 2011; Zhang et al., 2024). Evidence for pathological hypertrophy remains in low birth weight (LBW) neonatal and adolescent offspring (Briscoe et al., 2004; Chou & Chen, 2023; Darby et al., 2022; Wang et al., 2011), providing evidence that this is a programmed effect that may underpin the increased risk of CVD in adulthood.

PI delays the maturation of CMs as evidenced by the altered proportion of mononucleated *versus* binucleated CMs in late‐gestation and neonatal sheep hearts (Barooni et al., 2026; Botting et al., 2014; Bubb et al., 2007; Chang et al., 2024; Jonker, Kamna, et al., 2018; Morrison et al., 2007). The normal developmental surge in TH is known to promote this maturational process (Amanollahi, et al., 2025; Chattergoon et al., 2012; Chattergoon et al., 2023; Dimasi et al., 2023). The fetal secretion of cortisol directly influences the fetal thyroid axis, and in PI fetuses, there is an earlier and greater prepartum rise in plasma cortisol concentration (Brown et al., 2022; Jonker, Kamna, LoTurco, et al., 2018; Louey et al., 2007; Phillips et al., 1996; Sutherland et al., 2024). This earlier rise may influence TH production and deiodination with subsequent delays in CM maturational processes (Amanollahi, et al., 2025; Amanollahi et al., 2025; Chattergoon et al., 2012). In LBW lambs, TH concentrations in circulation (predominantly T4) are lower, specifically when the lamb is hypoglycaemic due to fasting (Cabello & Levieux, 1980, 1981; De Blasio et al., 2006; Ramos‐Nieves et al., 2020). However, evidence for PI altering the concentrations of cortisol and TH within fetal cardiac tissue is currently limited (Dimasi et al., 2024).

Operating in tandem with delayed CM maturation is a delay in the cardiometabolic profile with CMs maintaining immature features and failing to upregulate the cellular machinery needed to efficiently metabolize FAs via mitochondrial oxidative phosphorylation (OXPHOS) in late gestation (Amanollahi, et al., 2025; Chattergoon et al., 2012; Chattergoon et al., 2023). In early‐onset PI, key regulators of mitochondrial biogenesis and early signs of oxidative stress due to increased mitochondrial reactive oxygen species (ROS) production are evident by the end of the first trimester (Figueroa et al., 2017; Zhang et al., 2024). By late gestation, cardiac abundance of mitochondrial OXPHOS complexes, respiration, FA transporters, and the optimal redox ratio are reduced in two sheep models (Chang et al., 2024; Dimasi et al., 2021; Dimasi et al., 2024). Late PI can also decrease expression of FA metabolism genes and mitochondrial activity in the fetal heart (Drake et al., 2022; Gonzalez‐Tendero et al., 2013; Guitart‐Mampel et al., 2018; Simões et al., 2018). Signs of cardiometabolic dysfunction persist in LBW neonatal lambs and guinea pigs (Azman, et al., 2025; Darby et al., 2022; Wang et al., 2013). The less efficient FA metabolism observed across multiple models of PI is likely multifactorial, including reduced fetal availability of FAs, prevailing fetal hypoxaemia and a self‐perpetuating cycle of mitochondrial dysfunction that worsens as the insult continues.

Early‐onset PI reduces cardiac abundance of the insulin‐dependent glucose transporter (GLUT) 4 in late gestation (Botting et al., 2014; Dimasi et al., 2024; Tsirka et al., 2001); however, cardiac insulin sensitivity and markers of insulin‐stimulated glucose metabolism increase in late gestation due to PI, persisting in the LBW neonatal lamb (Barry et al., 2006; Barry et al., 2016; Wang et al., 2013). Nonetheless, these early origins of cardiac insulin resistance reflect the peripheral insulin‐resistant phenotypes present in late‐gestation sheep (Barry et al., 2016; Gatford et al., 2008; Limesand et al., 2012; Owens et al., 1989) and persist into adulthood (Camacho et al., 2017; De Blasio et al., 2007; Gatford et al., 2010; Owens et al., 2007; Wang et al., 2013). Associated with altered glucose metabolism is glycogen mishandling, with early‐onset PI increasing glycogen content in late gestation (Barry et al., 2006). In contrast, late‐onset PI reduces glycogen content, indicating an increase in glycogen breakdown to support glycolysis that normalizes shortly after birth in LBW offspring born to early‐ and late‐onset PI (Chou & Chen, 2023; Tsirka et al., 2001; Wang et al., 2013).

PI induces extracellular matrix (ECM) remodelling, including increased collagen content and genes in the late‐gestation heart that persist into adolescence and adulthood (Azman, et al., 2025; Briscoe et al., 2004; Darby et al., 2022; Thompson et al., 2013). Increased collagen deposition suggests fibrosis, which coincides with an increase in mechanical stiffness and ventricular thickness observed in sheep and rats (Alhama‐Riba et al., 2025; Dodson et al., 2017; Murotsuki et al., 1997; Rock et al., 2023). Fibrosis can result from hypertension, and indeed late‐onset PI induces hypertension in late‐gestation fetuses (Cock & Harding, 1997; Galan et al., 2005; Inocencio et al., 2020; Murotsuki et al., 1997) that is programmed and persists in offspring (Baserga et al., 2007; Black et al., 2012; Dasinger et al., 2016; Louey et al., 2000; Wlodek et al., 2008). Whereas early‐onset PI fetuses are often normotensive in late gestation (Drake et al., 2022; Dyer et al., 2009; Edwards et al., 1999; Regnault et al., 2007; Thompson et al., 2011) but maintain blood pressure differently, with evidence for disrupted cardiorespiratory control centre and hyperinnervation of the peripheral vasculature (Ahmadzadeh et al., 2024; Danielson et al., 2005; Darby et al., 2021; Oyang et al., 2023), which may predispose them to hypertension in later life.

The combination of diminished ATP yield due to metabolic inflexibility and a mechanically stiffer heart results in an altered contractile profile. Early and late‐onset PI exhibit alterations in markers of calcium (Ca^2+^) handling and sarcomere length, the CM contractile unit, in late gestation (Azman, Piscopo, Sutherland, et al., 2025; Dimasi et al., 2024; Torre et al., 2014). While some cardiovascular parameters remain normal in late gestation despite PI (Dimasi et al., 2024; Rock et al., 2023; Schipke et al., 2017; Torre et al., 2014), these fetal hearts overcompensate to maintain left ventricular cardiac output (LVCO), and immediately after birth, PI‐born fetuses can have lower LVCO (Polglase et al., 2016; Tare et al., 2012). Furthermore, functional recovery from secondary postnatal insults (ischaemia‐reperfusion (IR) injury) is impaired in preterm FGR lambs (Oyang et al., 2023). Cardiovascular structure and function continue to change in the weeks following birth, with evidence for reduced cardiac output and Ca^2+^ mishandling (Dodson et al., 2017; Rock et al., 2023; Rock et al., 2026; Wang et al., 2015) with reduced sarcomere length and shifts in myosin heavy chain (MYH) isoforms persisting in adulthood (Darby et al., 2022; Torre et al., 2014). However, PI is associated with fetal hypoxaemia and reduced nutrient availability. The impact of changes in nutrient availability, particularly glucose as the main fuel for the fetal heart, without hypoxaemia, on cardiac development is also important in understanding the mechanisms that underpin increased offspring CVD risk.

### Maternal nutrient restriction: the impact of low glucose and normal oxygen on fetal heart development

The impact of MNR on fetal body and heart weight is variable, with no or subtle decreases compared with PI (Fig. 3) (Bertossa, et al., 2025; Edwards & McMillen, 2002b; Poore et al., 2007). However, in some instances, when MNR occurs early in gestation during rapid placental development, dysfunction of the placenta can arise, leading to phenotypes similar to PI‐FGR, such as reduced fetal body and/or heart weight (Edwards & McMillen, 2002b; Elias et al., 2016; Pereira et al., 2021; Vonnahme et al., 2003; Woodall et al., 1996b). Indeed, similar to PI‐FGR, circulating IGF1 concentrations are reduced in MNR fetuses (Dong et al., 2008; Dwyer & Stickland, 1992; Osgerby et al., 2002; Woodall et al., 1996a) and remain decreased in early life and into adulthood (Hoffman et al., 2014; Smith et al., 2014; Woodall et al., 1996a). There is no evidence for changes in fetal cardiac IGF1 expression and abundance with early or late MNR (Bertossa et al., 2026; Darby et al., 2018a; Dong et al., 2005; Lie et al., 2013); however, compensatory increases in IGF1R expression and downstream cell cycle regulators (cyclins) have been reported in the fetal heart in mid‐ and late‐gestation sheep and rats (Dong et al., 2005; Han et al., 2004; Zouridis et al., 2021).

Early gestation MNR can reduce fetal heart weight and CM numbers, accompanied by an increase in markers of apoptosis in late‐gestation guinea pigs (Masoumy et al., 2018); however, this model of MNR resulted not only in fetal hypoglycaemia but also hypoxaemia similar to PI due to impaired placental development (Elias et al., 2017). Suggesting hypoglycaemia and hypoxaemia in combination lead to reduced CM endowment. Early gestation MNR also activates mTOR‐mediated autophagy pathways within the baboon fetal heart, suggesting CM cellular components are being recycled to maintain survival in late gestation (Lie et al., 2013; Muralimanoharan et al., 2017; Pereira et al., 2021). Early gestation MNR can increase the number of mononucleated CMs in late gestation guinea pigs (Masoumy et al., 2018); however, mixed results exist regarding whether binucleation is decreased (Lemley et al., 2012; Mazinani et al., 2022). Nonetheless, MNR results in larger CMs and increased molecular markers of pathological hypertrophy in late gestation that persist into adulthood (Bertossa, et al., 2026; Darby et al., 2018a; Kawamura et al., 2007; Lemley et al., 2012; Vonnahme et al., 2003), which is a similar outcome to PI.

In early‐ and late‐gestation MNR models, indices of ECM remodelling accompany hypertrophy, including increased collagen gene expression and content as well as ventricle wall thickness in the fetal heart (Darby et al., 2018a; Dong et al., 2005; Muralimanoharan et al., 2017; Vonnahme et al., 2003) that persist in MNR‐born LBW neonates into adulthood in baboons, sheep, guinea pigs and rats (Bertram et al., 2008; Kuo, Li, Huber, et al., 2017; Rodríguez‐Rodríguez et al., 2021; Xu, Williams, O'Brien, et al., 2006). Contributing to CM enlargement is fetal hypertension, which is associated with modified baroreflex and cardiac renin–angiotensin system function, with these changes persisting after birth and contributing to hypertension risk in adulthood (Aguilera‐Méndez et al., 2024; Bertram et al., 2008; Edwards & McMillen, 2001, 2002b; Gardner et al., 2004; Hawkins, Steyn, Ozaki, et al., 2000; Rodríguez‐Rodríguez et al., 2015; Woodall et al., 1996b). Although the occurrence of hypertension is similar to PI, there is less evidence for a role of SNS hyperinnervation in MNR than PI, which is likely driven by fetal hypoxaemia.

Much like PI, early‐ and late‐gestation onset MNR dysregulates the fetal hypothalamic–pituitary–adrenal (HPA) axis in late gestation, elevating plasma cortisol concentration and offspring to fibrosis, hypertrophy and hypertension (Begum et al., 2013; Bloomfield et al., 2003; Edwards & McMillen, 2001, 2002, a,b; Hawkins 2000; Li et al., 2013; Nathanielsz et al., 2020; Poore et al., 2010; Vonnahme et al., 2013). In contrast to PI, there is stronger evidence for early‐ and late‐onset MNR reducing circulating fetal THs, predominantly thyroxine (T_4_) concentrations in mid‐ and late gestation that persist in neonates and adolescents (Hoffman et al., 2014; Jones et al., 1990; Lingas et al., 1999; Steinhauser, Askelson, Hobbs, et al., 2021; Vonnahme et al., 2003). Also in contrast to PI, early and late MNR do not change cardiac tissue cortisol concentrations, expression of glucocorticoid receptor (GR), or cortisol‐converting enzymes in fetal and adult hearts (Bertossa,, et al., 2026; Bertossa, Darby, Holman, Meakin, et al., 2025; Darby et al., 2018a; Whorwood et al., 2001). However, MNR in late gestation can reduce cardiac tissue TH and expression of factors involved in TH signalling when assessed in late‐gestation baboons and neonatal rats (Aláez et al., 1992; Bertossa, et al., 2025; Zouridis et al., 2021). In the long term, MNR‐born offspring have reduced plasma TH concentrations and cardiac expression of genes required for TH uptake or conversion into adulthood (Ayala‐Moreno et al., 2013; Johnsen et al., 2013; Johnsen et al., 2018; Kamel et al., 2012; Konieczna et al., 2015).

TH deficits likely contribute to reports of immature cardiometabolic profiles. Periconceptional, early‐ and late‐onset MNR each alter lipid handling, reduce mitochondrial number, OXPHOS activity and abundance in mid‐ and late‐gestation sheep and baboon hearts (Bertossa, et al., 2026; Han et al., 2004; Muralimanoharan et al., 2017; Pereira et al., 2021; Pereira et al., 2023; Zouridis et al., 2021). These cardiometabolic changes are similar to PI; however, more evidence exists for early‐ or late‐gestation MNR reducing expression of key regulators of FA metabolism, mitochondrial DNA content and respiration in adulthood (Beauchamp, Thrush, Quizi, et al., 2015; Chan et al., 2009; Monedero Cobeta et al., 2024; Oliveira et al., 2017). There is also evidence for reduced myocardial glucose uptake and peripheral insulin resistance in baboons, sheep and rats postnatally (Beauchamp, Ghosh, Dysart, et al., 2015; Blondeau et al., 2001; Choi et al., 2011; Cripps et al., 2008; Hoffman et al., 2016; Poore et al., 2007).

Like PI, MNR leads to altered contractility profiles such that early and late‐onset MNR increases titin expression by mid–late gestation, and alters markers of Ca^2+^ handling in late gestation, which may in part relate to increased biventricular ejection fraction in sheep and baboons (Bertossa et al., 2026; Bertossa, et al., 2025; Cho et al., 2026; Darby et al., 2018a; Han et al., 2004; Zouridis et al., 2021). In MNR‐born offspring, no changes in cardiovascular parameters are observed in young adulthood (Bertram et al., 2008; Xu, Armstrong, Williams, et al., 2006). At the molecular level, there are shifts in expression of MYH isoforms, a decrease in contraction efficiency, ryanodine receptor abundance, and Ca^2+^ mishandling, indicative of a degree of diastolic dysfunction (Harvey et al., 2015; Xu, Armstrong, Williams, et al., 2006). With ageing, phenotypes consistent with biventricular diastolic and systolic dysfunction become evident in baboons and rat MNR offspring (Kuo, et al., 2017; Kuo, et al., 2017; Rodríguez‐Rodríguez et al., 2017; Zócalo et al., 2020).

Collectively, the evidence suggests that there are some differences in cardiac development in response to PI *versus* MNR and that the underlying mechanisms that lead to increased CVD risk may differ. Reduced CM endowment appears to be a response to hypoxaemia in combination with hypoglycaemia, and while hypercortisolaemia can occur in PI and MNR, reduced THs may be specific to fetal hypoglycaemia. CM hypertrophy and both mitochondrial and contractile dysfunction may arise regardless of the type of insult if it occurs during critical windows (i.e. early and late) of fetal cardiac development (Darby et al., 2020; Morrison, 2008).

### Fetal hypoxaemia: the impact of low oxygen and normal glucose on fetal heart development

Maternal hypoxia (MH) is more likely to reduce fetal body weight, like PI, which contrasts with MNR (∼20% on average across models, Fig. 3) (Allison, et al., 2016; Botting et al., 2020; Camm et al., 2010; Cuffe et al., 2014; Smith et al., 2022; Song et al., 2021; Williams et al., 2005; Xiao et al., 2000) and can reduce heart weight in rats and guinea pigs (Bae et al., 2003; Botting et al., 2020; Camm et al., 2010; Thompson et al., 2020; Xiao et al., 2000). By contrast, fetal anaemia that causes fetal hypoxaemia, has no impact on fetal body weight (Benjamin et al., 2017; Bernard et al., 2012; Davis et al., 2018; Jonker et al., 2010; Olson et al., 2006; Yang et al., 2008) and fetal heart weight is either increased or unaffected (Davis et al., 1996, 1999; Davis et al., 2018; Jonker et al., 2010; Olson et al., 2006). Indeed, differences in fetal outcomes depend on the origin of hypoxia, which can arise pre‐placentally (MH) or post‐placentally (fetal anaemia), impacting how the fetus responds (Kingdom & Kaufmann, 1997).

Early‐ and late‐gestation hypoxaemia can reduce markers of CM proliferation in early‐ and late‐gestation fetal hearts (Jonker, et al., 2015; Olson et al., 2006; Ream et al., 2008; Sun et al., 2019; Wendler et al., 2007); the latter results in reduced CM number in late gestation (Botting et al., 2014; Österman et al., 2015), which persists into adolescent guinea pigs (Botting et al., 2018). Similar to PI, early and late MH induce pro‐apoptotic cardiac responses in rats (Bae et al., 2003; Giussani et al., 2012; Romanowicz et al., 2021), which is in contrast to no changes in apoptosis in MNR (Bae et al., 2003; Ream et al., 2008; Tintu et al., 2009; Xiao et al., 2000). In adulthood, this pro‐apoptotic response to MH remains (Gusev et al., 2024; Li et al., 2003; Rueda‐Clausen et al., 2012) and thus, like PI, apoptosis may underlie the decrease in CM endowment.

Independent of the cause of hypoxaemia, fetal CMs show evidence for pathological hypertrophy persisting into adulthood (Bae et al., 2003; Ganguly et al., 2020; Jonker et al., 2010; Kumar et al., 2020; Li et al., 2004; Li et al., 2018; Paz et al., 2025; Wang et al., 2014). This hypertrophy response is observed across PI and MNR. CM enlargement may be secondary to reports of fetal hypertension that are associated with altered cardiovascular chemoreflex function that persists into adulthood (Botting et al., 2020; Brain et al., 2019; Gardner et al., 2002; Kamitomo et al., 1992). Moreover, indices of ECM remodelling are present in the fetal heart and persist into young and late adulthood in sheep, guinea pigs and rats (Botting et al., 2020; Evans et al., 2012; Li et al., 2018; Paz et al., 2025; Rueda‐Clausen et al., 2009; Tong et al., 2011; Wang et al., 2014; Xu, Williams, O'Brien, et al., 2006). Contributing to ECM remodelling is hypercortisolaemia due to a resetting of fetal HPA axis function in late gestation, also known to produce hypertension (Braems, 2003; Gagnon et al., 1994; Giussani et al., 2011; Green et al., 2000; Murotsuki et al., 1996). This cortisol overexposure may be responsible for a compensatory downregulation of cardiac GR protein abundance and mRNA expression in the fetal heart that persists into adulthood (Lv et al., 2019; Xue et al., 2011). In contrast to PI and MNR, no studies have reported how hypoxaemia alone affects cardiac glucocorticoid and TH concentrations.

Early‐ and late‐gestation hypoxaemia increase markers of oxidative stress in the fetal circulation and heart (Al‐Hasan et al., 2013; Botting et al., 2020; Giussani et al., 2012; Li et al., 2018; Spiroski et al., 2021), which coincides with increased antioxidant enzyme expression (Brain et al., 2019; Dimasi et al., 2024; Spiroski et al., 2021). Oxidative stress can induce cardiac infiltration of immune cells with increased expression of inflammatory genes (Davis et al., 2018; Gao et al., 2019; Mascio et al., 2005; Thompson et al., 2009; Tintu et al., 2009). Despite this, indices of oxidative stress remain in adolescence and adulthood (Evans et al., 2012; Giussani et al., 2012; Hellgren et al., 2021; Paz et al., 2025; Thompson et al., 2009). Likewise, markers of inflammation in the circulation and cardiac tissue persist into adulthood (Allison, et al., 2016; Gao et al., 2019; Gusev et al., 2024; Huang et al., 2019; Paz et al., 2025).

Some PI and MNR studies report that oxidative stress is present, likely owing to all conditions causing a degree of mitochondrial damage and increased mitochondrial‐derived ROS production. Indeed, early and late MH can reduce mitochondrial content and density and induce mitochondrial degradation, associated with altered fission and fusion properties, as well as reduced OXPHOS abundance, enzymatic activity, and mitochondrial respiratory efficiency in late‐gestation rats and guinea pigs (Al‐Hasan et al., 2013; Botting et al., 2020; Li et al., 2018; Smith et al., 2022; Song et al., 2021). High‐risk cardiometabolic phenotypes are programmed to persist into adolescence and adulthood in rats and guinea pigs (Al‐Hasan et al., 2014; Gao et al., 2019; Hashimoto et al., 2003; Hellgren et al., 2021; Thompson et al., 2018).

To compensate for low ATP yield and hypoxaemia, there is increased expression of genes required for glucose transport and glycolysis in the fetal and neonatal period (Fisher et al., 1982; Mascio et al., 2005; Ohtsuka & Gilbert, 1995; Ream et al., 2008; Romanowicz et al., 2021). Early and late MH can increase glucose storage as glycogen in the late‐gestation heart, likely due to increased cardiac glucose uptake (Tintu et al., 2009; Tong et al., 2026), suggesting that hypoxaemia may drive a similar response to early gestation PI (Barry et al., 2006). In late‐gestation hypoxaemia, glycogen metabolism and content are reduced, indicating rapid use of stored glucose and thus glycolytic dominance (Gao et al., 2019; Lewis et al., 1999). While cardiac metabolism appears normal in late adulthood, these hearts are sensitive to a secondary postnatal insult (IR injury) (Rueda‐Clausen et al., 2011; Xue & Zhang, 2009).

Collectively, this disrupted fetal cardiometabolic profile ultimately leads to poor ATP yield, negatively impacting cardiac function (Thompson et al., 2024). To counteract this and maintain O_2_ delivery to vital organs in light of hypoxaemia, fetal cardiac output is redistributed and myocardial blood flow increased, involving expansion of the coronary vasculature supplying the heart (Davis & Hohimer, 1991; Fisher et al., 1982; Martin et al., 1998; Mascio et al., 2005; Reller et al., 1992a, 1992b). This haemodynamic response is achieved by increasing circulating and cardiac hypoxia‐inducible factor (HIF)‐1α, vascular endothelial growth factor and vasodilators like nitric oxide synthase expression (Brain et al., 2019; Mascio et al., 2005; Patterson et al., 2012; Romanowicz et al., 2021; Thompson et al., 2009). These factors mediate angiogenesis of myocardial capillaries, alterations in capillary ultrastructure (size and density) and increased shunting of blood through the ductus venosus toward the fetal heart and brain (Jonker et al., 2010, 2011; Mascio et al., 2005; Tchirikov et al., 1998). Aiding this fetal haemodynamic response to chronic hypoxaemia is sympathetic hyperreactivity of the peripheral vasculature, increased sympathetic responsiveness to β‐adrenergic stimulation and increased β‐adrenergic receptor (βAR) number, density and mRNA expression (Bae et al., 2003; Browne et al., 1997b; Hutchinson et al., 2018; Kamitomo et al., 1995; Lindgren et al., 2011; Rouwet et al., 2002). The impact of hypoxaemia on fetal sympathetic activity is often reflected by increased fetal heart rate variability (Frasch et al., 2020; Gagnon et al., 1996; Lear et al., 2024; Shaw et al., 2018). Many of the above indices of cardiac sympathetic dominance persist into adulthood (Giussani et al., 2012; Gusev et al., 2024; Hauton & Ousley, 2009; Li et al., 2003; Lock et al., 2024). These haemodynamic and sympathetic adaptations are more often features of PI than of MNR, highlighting fetal hypoxaemia rather than hypoglycaemia as the primary driver.

The increased sympathetic activity is also reflected by an increase in cardiac markers of Ca^2+^ cycling in late gestation due to chronic hypoxaemia (Browne et al., 1997a; Gilbert, 1998; Niu et al., 2018; Spiroski et al., 2021). However, indices of contractile dysfunction are evident in the late‐gestation and neonatal heart due to hypoxaemia (Botting et al., 2020; Jonker, Giraud, Espinoza, et al., 2015; Kamitomo et al., 1992; Onishi et al., 2004; Tintu et al., 2009). This dysfunction may not arise solely from Ca^2+^ mishandling, as other mechanisms related to the structural remodelling of the contractile unit may contribute (Gao et al., 2019; Hashimoto et al., 2003; Romanowicz et al., 2021; Tintu et al., 2009; Xu, Williams, O'Brien, et al., 2006). In adulthood, offspring continue to have contractile dysfunction due to programmed Ca^2+^ mishandling and diastolic dysfunction (Lock et al., 2024; Onishi et al., 2003; Rueda‐Clausen et al., 2009; Thompson et al., 2018; Xu, Williams, O'Brien, et al., 2006) while functional recovery from a secondary postnatal insult (e.g. IR injury) is reduced (Hauton & Ousley, 2009; Li et al., 2003; Shah et al., 2016; Xu, Williams, O'Brien, et al., 2006). Regardless of the pregnancy complications or substrate deficiency (oxygen and/or glucose), contractile dysfunction is a common endpoint; however, the upstream mechanisms leading to this may differ.

### The differential effect of restricted substrates on the fetal right and left ventricle

**Figure 4:**
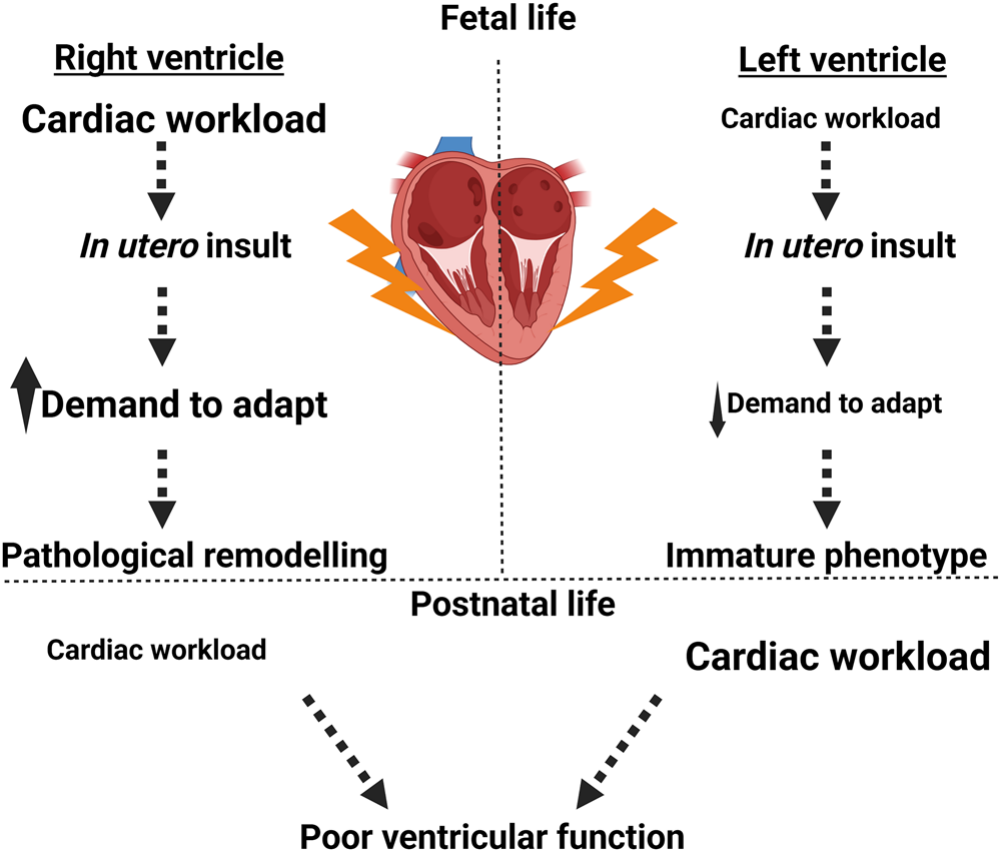
Differences between the fetal right ventricle (RV) and left ventricle (LV) baseline workloads and demands to adapt to *in utero* insults The fetal RV contributes to ∼60% of the combined ventricular output and experiences an increased demand to adapt to reduced substrate availability *in utero*. By comparison, the fetal LV has less demand to adapt, which may also serve to be a disadvantage when it becomes the dominant ventricle after birth.

The fetal left ventricle (LV) and right ventricle (RV) originate from different embryological regions with their CMs differing in terms of morphology, turnover rate, metabolic properties and myofibre properties (sarcomere length) as well as maturation status (Chang et al., 2024; Jonker, Kamna, LoTurco, et al., 2018; Kim et al., 2014; Morrison et al., 2007). These differences are necessary to allow each ventricle to meet its functional requirements, whereby the unique characteristics of the fetal circulation, including the presence of the ductus arteriosus, lead to a higher workload for RV than LV to support systemic circulation (∼66% of combined ventricular output) (Edelstone & Rudolph, 1979). In the face of reduced substrate availability, there may be an increased demand for adaptation of the RV to support increased workload (Fig. 4) (Barbera et al., 2000). Indeed, in MH FGR fetuses, RV dysfunction becomes more pronounced than LV dysfunction (Gilbert, 1998; Onishi et al., 2003, 2004). To compensate, the RV exhibits an increase in βAR responsiveness, βAR density and a greater shift towards MYH7 for more energy‐efficient contractions (Browne et al., 1997a; Kamitomo et al., 1995). The RV elicits greater cellular adaptations to increase local oxygen delivery, such as increased angiogenesis, capillary dimension/density and adenosine signalling compared with the LV, in which these factors may be downregulated (Botting et al., 2014; Davis et al., 2018; Hauton & Ousley, 2009; Mascio et al., 2005; Schipke et al., 2017). However, these adaptations can increase RV volume loading and pressure, partly explaining the CM enlargement observed only, or to a greater extent, in the RV than LV (Bubb et al., 2007; Hashimoto et al., 2003; Jonker et al., 2010; Lock et al., 2024; Master et al., 2014) as well as other indices of pathological hypertrophy (Darby et al., 2018b; Dong et al., 2005; Murotsuki et al., 1997; Paz et al., 2025). ECM remodelling also occurs to a greater extent in the RV in response to hypoxaemia and/or hypoglycaemia, including collagen‐related genes, collagen content and greater wall volumes and thickness compared with the LV where these are often reduced or unchanged (Bertossa, et al., 2026; Darby et al., 2018a; Jonker, et al., 2015; Kuo, Li, Li, et al., 2017; Murotsuki et al., 1997; Rock et al., 2023; Schipke et al., 2017; Thompson et al., 2013; Tong et al., 2011; Wendler et al., 2007). This ventricle‐specific ECM remodelling is consistent with human FGR neonates (Chen et al., 2024; Patey et al., 2019; Rodriguez‐Guerineau et al., 2018). Even after birth, reduced RV contractility is more pronounced, and mitochondrial function remains more sensitive to hypoxaemia than the LV (Keenaghan et al., 2016; Kuo, Li, Huber, et al., 2017).

The lower workload and reduced demand for LV adaptation may be disadvantageous (Fig. 4). For example, increased CM apoptosis and/or gene signatures consistent with apoptotic priming, paralleled by reduced CM numbers and markers of cell cycle activity, occur only in the late‐gestation LV or to a greater extent in the LV than the RV (Botting et al., 2014; Jonker, et al., 2015; Mattern et al., 2023; Schipke et al., 2017; Tintu et al., 2009). CMs in the LV display an immature phenotype that may be absent in the RV, including reduced binucleation and mitochondrial content, a shift in GLUT1/4 abundance, as well as contractile abnormalities (Bernard et al., 2012; Botting et al., 2014; Bubb et al., 2007; Chang et al., 2024). These data highlight that ventricle‐specific developmental programming should be taken into consideration when conducting studies of fetal or postnatal cardiovascular physiology. In terms of postnatal implications, the preexisting remodelling of the RV may set the stage for further functional decline in conditions complicated by pulmonary hypertension, common in preterm FGR infants. The LV likely faces additional burdens when it must adapt to the sudden transition to being the dominant ventricle at birth.

### Males are more sensitive than females, despite a comparable *in utero* environment

**Figure 5:**
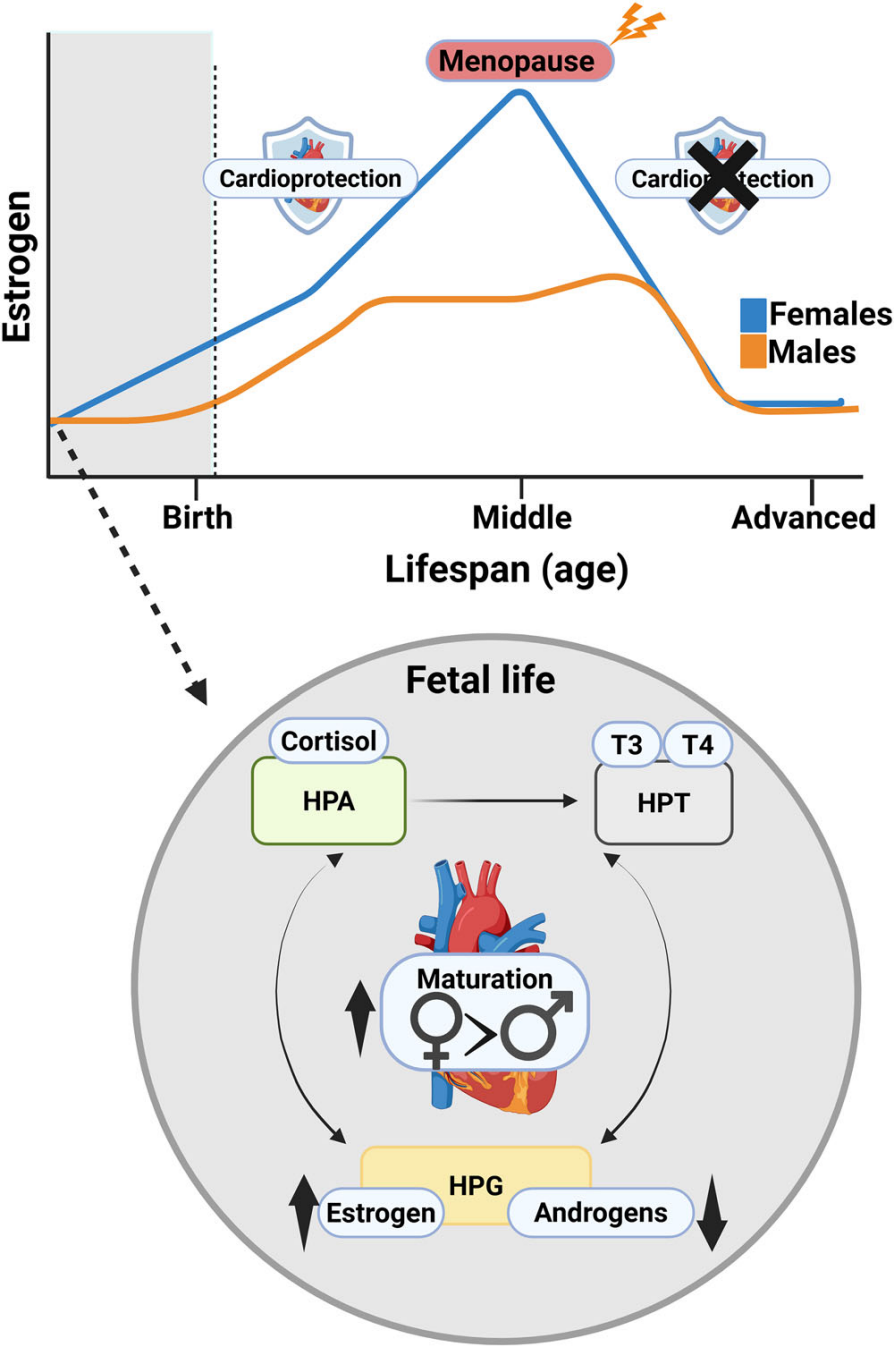
The complex interaction between fetal endocrine axes results in different maturation and cardioprotective properties between male and female hearts across the lifespan There is crosstalk between the hypothalamic–pituitary–gonadal (HPG) axis, which produces oestrogen and testosterone, the hypothalamic–pituitary–adrenal (HPA) axis, which produces cortisol, and the hypothalamic–pituitary–thyroid (HPT) axis, which produces thyroid hormones triiodothyronine (T_3_) and thyroxine (T_4_), leading to differences in heart maturation status between sexes. In postnatal life, oestrogen concentrations continue to increase throughout early development, providing more cardioprotection for females than males. The onset of ageing and menopause causes a drop in oestrogen, dampening the oestrogen cardioprotective effects. Blue line = females; Orange line = males.

To comprehensively understand how deficits in oxygen and nutrients like glucose impact fetal heart development, it is important to consider the sex of the fetus under investigation. Under normal conditions, sex differences related to heart structure (cell type, composition, total CM numbers), metabolism, contractility and microRNA profiles exist (Chang et al., 2024; Darby et al., 2022; Dimasi et al., 2024; Ghnenis et al., 2022; Muralimanoharan et al., 2017). For example, the number of mature CMs is greater in females than in males in late gestation (Jonker, et al., 2018; Lumbers et al., 2009). This is partly attributed to the hypothalamic–pituitary–gonadal (HPG) axis that produces steroid hormones (predominantly oestrogen in females and androgens in males). These hormones exert distinct effects on cardiac health across the life course, such that oestrogens provide relative cardioprotection compared with androgens (Fig. 5) (McClements et al., 2025; Meakin et al., 2021). While gonad function is immature in fetal life and both males and females have similar circulating oestrogen and androgen concentrations, oestrogen receptors are more abundant in female than male hearts, which may enhance CM maturation (Chen et al., 2013; Patterson et al., 2012). In contrast, androgen concentrations (i.e. testosterone) are greater in male hearts, and administration of synthetic testosterone can induce FGR and delay indices of CM maturation to a greater extent in male than female fetuses (Ghnenis et al., 2022; Jonker, et al., 2018). This HPG axis also interacts with the HPA and hypothalamic–pituitary–thyroid axes, influencing the timing of activation and function that results in plasma cortisol and TH concentrations differing in fetal and neonatal circulation between sexes (Fig. 5) (Ayala‐Moreno et al., 2013; Camacho et al., 2012; Edwards & McMillen, 2002a; Johnsen et al., 2018; Vonnahme et al., 2013).

These sex differences lay the foundation for sexually dimorphic programming in response to reduced substrate availability, whereby male fetuses generally prioritize growth compared with females that prioritize adaptive responses in the placenta and fetal tissues (Clifton et al., 2012; Cox et al., 2013; Eriksson et al., 2010; Meakin et al., 2021). Indeed, pathological remodelling predominates, including hypertrophy, fibrosis and autophagy in late‐gestation male non‐human primate hearts exposed to MNR compared with females (Muralimanoharan et al., 2017; Pereira et al., 2021). In MNR‐born rodent offspring, CM proliferation is reduced in only male neonatal hearts and the remaining CMs are more likely to undergo pathological remodelling in young and late adulthood, regardless of the cause of FGR (Darby et al., 2022; Reyes et al., 2018; Rueda‐Clausen et al., 2012; Xu, Williams, O'Brien, et al., 2006). These high‐risk fetal cardiac phenotypes are more often present in males than females, or are absent in females, and may relate to male‐predominant hypertension reported in a range of FGR models in sheep, guinea pigs and rats (Alexander, 2003; Bennet et al., 2007; Ozaki et al., 2001; Rodríguez‐Rodríguez et al., 2017; Wlodek et al., 2008).

In late gestation, males exposed to either MNR or MH display worsened cardiac mitochondrial morphology and dynamics in baboons, guinea pigs and rats (Al‐Hasan et al., 2013; Pereira et al., 2021; Song et al., 2021; Thompson et al., 2024). Male fetuses also display a greater cardiac glycolytic dominance and Ca^2+^ mishandling in sheep and baboons (Bertossa, et al., 2025; Dimasi et al., 2024). The male disadvantages in fetal life would likely program for poor outcomes after birth.

Indeed, high‐risk metabolic phenotypes, including reduced mitochondrial respiration, reduced OXPHOS abundance and activity, and reduced mitochondrial FA transporters, are more likely to persist through adolescence into adulthood in LBW‐born males compared with females in rats and guinea pigs (Al‐Hasan et al., 2014; Botting et al., 2018; Darby et al., 2022; Gao et al., 2019). LBW‐born males are more likely to display Ca^2+^ mishandling and contractile dysfunction across the life course in these same species (Harvey et al., 2015; Rodríguez‐Rodríguez et al., 2022; Rueda‐Clausen et al., 2009; Rueda‐Clausen et al., 2011; Zouridis et al., 2021). LBW males also display insulin‐resistant phenotypes into adulthood that may be absent in females (Gatford et al., 2008; Owens et al., 2007; Smith et al., 2010). Moreover, reduced cardiac GR expression in fetal hearts persists into adulthood in male rats, possibly programmed due to greater cortisol exposure in fetal life (Giussani et al., 2011; Lv et al., 2019; Xue et al., 2011).

Oestrogen remains unchanged in fetal life despite MNR exposure, allowing a degree of cardioprotection in early developmental stages; however, oestrogen becomes dampened with age and particularly with the onset of menopause, with an earlier fall in oestrogen in MNR‐born female adolescent offspring (Fig. 5) (Franco Mdo et al., 2002; Vonnahme et al., 2003). This dampening coincides with reports of increased circulating and cardiac tissue cortisol due to HPA hyperactivity in adolescent and adult females, but not males, exposed to reduced substrate supply *in utero* (Amanollahi et al., 2025; Botting et al., 2018; Brown et al., 2022; Camacho et al., 2012; Nathanielsz et al., 2020; Poore et al., 2010; Rae et al., 2002). With advanced age, poor phenotypes emerge in both sexes, including hypertension and insulin resistance (Baserga et al., 2007; Choi et al., 2011). In contrast, MNR increases circulating testosterone concentrations in male fetuses followed by a postnatal decline that is linked to the predominantly male hypertension across the life course (Rae et al., 2002). Nonetheless, while poor cardiac phenotypes may emerge in both sexes with advanced age, where feasible, any study of fetal or postnatal physiology should aim to integrate sex as a biological variable, taking into account the developmental life stage of that species (Becker & Ahmed, 2025; Lindsey et al., 2021; Zucker et al., 2022).

## Conclusion

During a complicated pregnancy, the development of the fetal heart is sensitive to changes in oxygen and glucose availability, exerting distinct yet synergistic roles in shaping cardiac phenotypes. When substrates are restricted across developmental windows that are vulnerable to change (rapid growth and differentiation in early gestation or structural maturation in late gestation), high‐risk phenotypes emerge regardless of the specific substrates at play. Adding another layer of complexity is that high‐risk cardiac phenotypes are more prevalent in males than females, likely mediated by the role of oestrogens and androgens in fetal life. Compounding this further is the unique adaptive pattern between the ventricles in the face of reduced substrate availability, as the RV outpaces the LV, necessitating different demands for adaptation. The combination of these factors underscores the need for personalized interventions that account for the timing, duration, severity and type of exposure, the sex of the fetus, and the differential responses of the ventricles. The interplay between these factors highlights the complexity of fetal cardiac programming. In fact, the level of complexity extends beyond what this review sought to simplify, since we focused on glucose, the main energy source in fetal life and the most characterized nutrient deficiency; however, other nutrients also play roles and they require further disentangling. Nonetheless, drawing conclusions on the effects of PI, MNR and hypoxia on cardiac outcomes across studies proved to be difficult, as outcomes are influenced by not only substrates, sex and ventricles examined, but also other experimental design factors including species and litter size, models of FGR used and outcome measures. Despite these complexities, this review highlights promising mechanistic pathways and potential as candidate targets for intervention that could protect against the fetal origins of CVD.

## Additional information

### Competing interests

None declared.

### Author contributions

M.R.B. and J.L.M. were responsible for the conception and design of the article. All authors were involved in the interpretation of the data, drafted the article, contributed to the final version and approved the manuscript submission.

### Funding

J.L.M. was funded by an Australian Research Council Future Fellowship (Level 3; FT170100431) and an NHMRC Investigator Grant (Leader 2; GNT2041967). J.R.T.D. was funded by a University of South Australia Enterprise Postdoctoral Fellowship. A.S.M. was funded by a National Heart Foundation Postdoctoral Fellowship (108157‐2024_PDF). M.R.B. was funded by an Australian Research Training Program Scholarship.

## Supporting information


Peer Review History
