# Supplementary material for: Insights from animal models: Dissecting the independent roles of oxygen and nutrients in the fetal origins of cardiovascular disease
Source: J Physiol. 2026 Mar 11;604(16):6704–30. doi: 10.1113/JP289670 (PMC13481021; doi:10.1113/JP289670)
Supplement: Supplementary file 1 [file TJP-604-6704-s001.pdf]

The Journal of Physiology

Insights from animal models: Dissecting the independent roles of oxygen and nutrients in the fetal origins of cardiovascular disease

Melanie Rose Bertossa, Jack RT Darby, Ashley Meakin, Mitchell C Lock, Steven KS Cho, Christopher K Macgowan, Mike Seed, Michael D Wiese, and Janna L Morrison
DOI: 10.1113/JP289670

Corresponding author(s): Janna Morrison (janna.morrison@adelaide.edu.au)

The referees have opted to remain anonymous.

**Review Timeline:**		
	Submission Date:	14-Aug-2025
	Editorial Decision:	17-Sep-2025
	Revision Received:	27-Jan-2026
	Accepted:	11-Feb-2026

Senior Editor: Laura Bennet

Reviewing Editor: Christopher Lear

Transaction Report:

(Note: With the exception of the correction of typographical or spelling errors that could be a source of ambiguity, letters and reports are not edited. Depending on transfer agreements, referee reports obtained elsewhere may or may not be included in this compilation. Referee reports are anonymous unless the Referee chooses to sign their reports.)

1st Editorial Decision 17-Sep-2025

Dear Janna,

Re: JP-TR-2025-289670 "**Insights from animal models: Dissecting the independent roles of oxygen and nutrients in the fetal origins of cardiovascular disease** " by Melanie Rose Bertossa, Jack RT Darby, Ashley S Meakin, Mitchell C Lock, Steven KS Cho, Christopher K Macgowan, Mike Seed, Michael D Wiese, and Janna L Morrison

Thank you for submitting your manuscript to The Journal of Physiology. It has been assessed by a Reviewing Editor and by 2 expert referees and we are pleased to tell you that it is potentially acceptable for publication following satisfactory major revision.

The referee reports are copied at the end of this email.
Please address all the points raised and incorporate all requested revisions or explain in your Response to Referees why a change has not been made. We hope you will find the comments helpful and that you will be able to return your revised manuscript within 9 months. If your article is for a Special Issue, please note that we require your revised version within 2 months (rather than 9 months) in order to keep the Special issue on track. If you require longer than this, please contact journal staff: jp@physoc.org. Please note that this letter does not constitute a guarantee for acceptance of your revised manuscript.

ABSTRACT FIGURES: Authors are expected to use The Journal's premium BioRender account to create/redraw their Abstract Figures. Information on how to access this account is here:
https://physoc.onlinelibrary.wiley.com/journal/14697793/biorender-access.
This will enable Authors to create and download high-resolution figures. If authors have used the free BioRender service, they can use the instructions provided in the link above to download a high-resolution version suitable for publication.
The link provided should only be used for the purposes of this submission. Authors will be charged for figures created on this account if they are not related to this manuscript submission.

Your revised manuscript should be submitted online using the link in your Author Tasks: Link Not Available. This link is accessible via your account as Corresponding Author; it is not available to your co-authors. If this presents a problem, please contact journal staff (jp@physoc.org). Image files from the previous version are retained on the system. Please ensure you replace or remove any files that are being revised.
If you do not wish to submit a revised version of your manuscript, you must inform our journal staff (jp@physoc.org) or reply to this email to request withdrawal. Please note that a manuscript must be formally withdrawn from the peer review process at one journal before it may be submitted to another journal.

REVISION CHECKLIST:
Check that your Methods section conforms to journal policy: https://jp.msubmit.net/cgi-bin/main.plex?form_type=display_requirements#methods.

Check that data presented conforms to the statistics policy: https://jp.msubmit.net/cgi-bin/main.plex?form_type=display_requirements#statistics.

Upload a full Response to Referees file. To create your 'Response to Referees': copy all the reports, including any comments from the Senior and Reviewing Editors, into a Microsoft Word, or similar, file and respond to each point, using font or background colour to distinguish comments and responses and upload as the required file type.

Please upload two versions of your manuscript text: one with all relevant changes highlighted and one clean version with no changes tracked. The manuscript file should include all tables and figure legends, but each figure/graph should be uploaded as separate, high-resolution files.

You may also upload:
- 'Potential Cover Art' for consideration as the issue's cover image
- Appropriate Supporting Information (Video, audio or data set: see https://jp.msubmit.net/cgi-bin/main.plex?form_type=display_requirements#supp).

IMPORTANT POINTS TO NOTE WHEN REVISING YOUR MANUSCRIPT:
LANGUAGE EDITING AND SUPPORT FOR PUBLICATION: If you would like help with English language editing, or other article preparation support, Wiley Editing Services offers expert help, including English Language Editing, as well as translation, manuscript formatting, and figure formatting at www.wileyauthors.com/eeo/preparation. You can also find resources for Preparing Your Article for general guidance about writing and preparing your manuscript at www.wileyauthors.com/eeo/prepresources.

TRANSPARENT PEER REVIEW POLICY: To improve the transparency of its peer review process, The Journal of Physiology publishes online as supporting information the peer review history of all articles accepted for publication. Readers will have access to decision letters, including Editors' comments and referee reports, for each version of the manuscript, as well as any author responses to peer review comments. Referees can decide whether or not they wish to be named on the peer review history document.

We look forward to receiving your revised submission.
If you have any queries, please reply to this email and we will be pleased to advise.

Best wishes,

Laura Bennet
Senior Editor
The Journal of Physiology

REQUIRED ITEMS

- Please include an Abstract Figure file, as well as the Figure Legend text within the main article file. The Abstract Figure is a piece of artwork designed to give readers an immediate understanding of the Review Article and should summarise the main conclusions. If possible, the image should be easily 'readable' from left to right or top to bottom. It should show the physiological relevance of the Review so readers can assess the importance and content of the article. Abstract Figures should not merely recapitulate other figures in the Review. Please try to keep the diagram as simple as possible and without superfluous information that may distract from the main conclusion of the Review. Abstract Figures must be provided by authors no later than the revised manuscript stage and should be uploaded as a separate file during online submission labelled as File Type 'Abstract Figure'. Please ensure that you include the figure legend in the main article file. All Abstract Figures will be sent to a professional illustrator for redrawing and you may be asked to approve the redrawn figure before your paper is accepted.

- Please upload separate high quality figure files via the submission form.

- Author profile(s) must be uploaded via the submission form. Authors should submit a short biography (no more than 100 words for one author or 150 words in total for two authors) and a portrait photograph of the two leading authors on the paper. These should be uploaded and clearly labelled together in a Word document with the revised version of the manuscript. Any standard image format for the photograph is acceptable, but the resolution should be at least 300 DPI and preferably more. A group photograph of all authors is also acceptable, providing the biography for the whole group does not exceed 150 words.

EDITOR COMMENTS

Reviewing Editor:

Thank you for submitting your detailed review to the Journal. The authors are to be commended on undertaking such a difficult and detailed review. Nonetheless, the reviewers have highlighted some inconsistencies and issues that need to be addressed. Particular attention is needed in regards to the correct reporting of nutrient restriction vs hypoglycemia relative to study design as requested by reviewer 1. As suggested by reviewer 2, the review would be strengthened by greater critical synthesis of the literature where inconsistencies are apparent across studies - these insights will be particularly important for our readers.

The major issue unfortunately is the article's length, reaching over 11,000 words. JP typically commissions TR which are 2-4,000 words. The nature of this topic, and the novelty of consolidating this information does warrant a higher limit, but the authors still need to reduce the length considerably. Please address where it is possible to streamline information, reduce repetition and consolidate sections. The reviewers comments will help in this regard, but the authors should additionally consider whether the sections on biomarkers and intervention (which constitute around 2000 words) and associated figures are necessary to the key aims of this review, or if they can be removed to prioritise the word count for the preceding discussion.

Please also see 'Required Items' above.

REFEREE COMMENTS

Referee #1:

This invited review addresses the consequences of fetal oxygen and glucose deficiency on cardiovascular development, primarily through insights gained from animal models. The authors effectively highlight the complexity of disentangling the individual and combined effects of oxygen and glucose deprivation on the fetal heart. The stated goal to tease out the independent and synergistic contributions of these two critical substrates is timely and meaningful, with potential to inform our understanding of how specific oxidative substrate deprivation shapes lifelong cardiovascular health.

Overall, the review is comprehensive and provides a well organized discussion of nutrient and oxygen deprivation in fetal cardiovascular development. However, some important conceptual issues need clarification before the manuscript is suitable for publication. Most notably, the frequent conflation of fetal hypoglycemia with global maternal nutrient restriction complicates the interpretation of the literature and the conclusions drawn. Hypoglycemia and MNR are not interchangeable, and attributing the cardiovascular consequences of MNR solely to hypoglycemia is not accurate. This issue should be addressed carefully throughout the text, figures, and graphical abstract.

Major comments:

Distinction between FGR and SGA

The review at times uses "fetal growth restriction FGR" and "small for gestational age SGA" interchangeably. These conditions are not synonymous. SGA infants can be constitutionally small but healthy, while some FGR infants may be above the 10th percentile yet still pathologically growth restricted. A clear distinction between FGR and SGA would improve the precision of the review, particularly since this conflation is common in the broader literature.

Conflation of nutrient restriction and hypoglycemia

The focus on hypoglycemia and its cardiovascular consequences is appropriate and consistent with the stated aim of the review. However, many of the studies cited involve global maternal nutrient restriction, which cannot be equated with isolated fetal hypoglycemia. MNR typically results in multiple deficits such as amino acids and lipids, and attributing downstream cardiovascular effects solely to low glucose oversimplifies the biology. Greater emphasis on nutrient specific placental knockout models would strengthen this point. The graphical abstract and several figures for example Figures 1, 3, 4, 5, 6 should be revised to accurately reflect global nutrient deficiency rather than labeling these models as low glucose.

Placental and oxygen transfer considerations

The authors acknowledge that early MNR can impair placental development and oxygen transfer, but this issue could be addressed more thoroughly. For example, comparisons with placental insufficiency induced IUGR PI IUGR, which involves reduced transfer of both oxygen and multiple nutrients, may provide useful context. Additionally, distinguishing between pre placental, placental, and post placental hypoxia see Kingdom and Kaufmann, 1997 PMID: 9364596 would add nuance, as these conditions produce distinct maternal and fetal phenotypes with potentially different cardiovascular outcomes.

Minor comments:

Figure 1: The color coded bubbles on the fetal heart are confusing and should be clarified with a legend or key, similar to the explanatory boxes below.

Figures 3, 4, 5, 6: As noted above, these should not depict MNR effects solely as low glucose. Depending on the model, animals may fall under the combined oxygen and glucose deprivation category.

Figures 8 and 9: While interesting, these figures do not directly align with the stated focus on glucose versus oxygen deprivation, as they address more general issues of sex differences and therapeutic approaches in FGR. The authors may wish to clarify their relevance or adjust emphasis.

Line 115: Specify "non human primates" rather than simply "primates."

Line 417 to 420: Expand on the distinction between different forms of hypoxia pre, placental, post placental. See Kingdom and Kaufmann, 1997 PMID: 9364596. This is important as the consequences to mom and baby differ based on which compartments the hypoxia is impacting.

Summary:

This review is thorough, well written, and addresses an important topic with strong potential impact. However, improved precision in terminology and interpretation particularly regarding distinctions between hypoglycemia, MNR, and FGR versus SGA will be critical to ensuring clarity and accuracy. Addressing these issues will significantly enhance the strength and reliability of the manuscript.

Referee #2:

This comprehensive review by Bertossa et al. provides a detailed exploration of how placental insufficiency, maternal nutrient restriction (MNR), and fetal hypoxemia individually and collectively influence the fetal origins of cardiovascular disease. A key strength of the manuscript is its thorough analysis of cardiac programming across multiple domains, including growth, hormonal regulation, metabolism, contractility, and cardiac structure. The review offers a unique contribution by integrating an in-depth discussion of various animal models and the impact of developmental timing on cardiac outcomes. The paper also effectively addresses sexual dimorphism and ventricle-specific responses, highlighting differential right vs. left ventricular responses under conditions of nutrient and/or oxygen restriction. Despite substantial content and valuable insight, several areas require attention.

Primary concerns:

Writing is overly dense and could benefit from more selective focus and a clearer synthesis of key messages. For example, there is redundancy and repetition that cause the reader to lose essential points being made by the authors. One instance would be the discussion of mitochondrial dysfunction in all three conditions (PI, MNR and hypoxemia) being discussed in a nearly identical manner (lines 235-252, 368-378, 460-470); it seems this could be better integrated to reduce repetition and improve flow. Similar issues are present for several other concepts and require attention to enhance clarity and impact.

Figures. While the figures are informative, these (particularly Figures 4-6) are overcrowded and could be streamlined to distill the complex information into a more easily digestible form. I realize that the amount of information being visualized is quite large, but I did not find the current presentation in these 3 figures in particular to be effective.

The review includes what may be viewed as an excessive proportion of self-citations, which raises concerns about selection bias and whether the review adequately represents the broader literature. Please ensure that all references are necessary (self-citations and otherwise).

Limited critical analysis. The review does a commendable job of summarizing existing literature but lacks a sufficient critical evaluation or discussion of contradictory findings; this somewhat restricts its utility as an objective evidence base for the field. Just one example, lines 277-283: "Some fetuses remain normotensive" despite PI, but then states "However, these fetuses may be maintaining blood pressure differently" with no follow-up for why this may be the case; no discussion of why 30-50% of studies show no HTN effect or methodological differences that might explain discrepancies. Animal model limitations are noted but not followed by how these limitations affect conclusions or translational relevance; this should be evaluated more fully given the focus of the review on animal models. Essentially, there are many missed opportunities to take the nice synthesis of existing literature a step further by reconciling conflicting or mixed findings.

Translation to humans: A more detailed discussion of risks/limitations/challenges to the translation of findings to humans is required.

Minor: Inconsistent abbreviation use. Sometimes cardiomyocyte is spelled out, other places it is abbreviated as CM; same with FGR (sometimes "growth-restricted") and MNR (sometimes "nutrient restriction"). Consider using fewer abbreviations and/or providing an abbreviations table for the manuscript.

Overall, this is a well-researched and ambitious review that addresses a critical topic in the developmental programming of cardiovascular disease. Revisions are necessary to improve its clarity and enhance critical analysis. I encourage the authors to refine and condense the manuscript to maximize the impact and accessibility of the important points and information they want to convey.

END OF COMMENTS

EDITOR COMMENTS

Reviewing Editor:

Thank you for submitting your detailed review to the Journal. The authors are to be commended on undertaking such a difficult and detailed review. Nonetheless, the reviewers have highlighted some inconsistencies and issues that need to be addressed. Particular attention is needed in regard to the correct reporting of nutrient restriction vs hypoglycemia relative to study design as requested by reviewer 1. As suggested by reviewer 2, the review would be strengthened by greater critical synthesis of the literature where inconsistencies are apparent across studies - these insights will be particularly important for our readers.

The major issue unfortunately is the article's length, reaching over 11,000 words. JP typically commissions TR which are 2-4,000 words. The nature of this topic, and the novelty of consolidating this information does warrant a higher limit, but the authors still need to reduce the length considerably. Please address where it is possible to streamline information, reduce repetition and consolidate sections. The reviewers comments will help in this regard, but the authors should additionally consider whether the sections on biomarkers and intervention (which constitute around 2000 words) and associated figures are necessary to the key aims of this review, or if they can be removed to prioritise the word count for the preceding discussion.

We thank the reviewing editor for granting a higher word limit for this review. This is a comprehensive review. However, we acknowledge that appropriate cuts are needed to streamline our message to readers more effectively. As such, we hope that the total accumulated cut of ~3,000 words will be satisfactory whilst still conserving a comprehensive coverage of the breadth of work in this field.

REFEREE COMMENTS

Referee #1:
This invited review addresses the consequences of fetal oxygen and glucose deficiency on cardiovascular development, primarily through insights gained from animal models. The authors effectively highlight the complexity of disentangling the individual and combined effects of oxygen and glucose deprivation on the fetal heart. The stated goal to tease out the independent and synergistic contributions of these two critical substrates is timely and meaningful, with potential to inform our understanding of how specific oxidative substrate deprivation shapes lifelong cardiovascular health. Overall, the review is comprehensive and provides a well organized discussion of nutrient and oxygen deprivation in fetal cardiovascular development. However, some important conceptual issues need clarification before the manuscript is suitable for publication. Most notably, the frequent conflation of fetal hypoglycemia with global maternal nutrient restriction complicates the interpretation of the literature and the conclusions drawn. Hypoglycemia and MNR are not interchangeable, and attributing the cardiovascular consequences of MNR solely to hypoglycemia is not accurate. This issue should be addressed carefully throughout the text, figures, and graphical abstract.

Major comments:

Distinction between FGR and SGA

The review at times uses "fetal growth restriction FGR" and "small for gestational age SGA" interchangeably. These conditions are not synonymous. SGA infants can be constitutionally small but healthy, while some FGR infants may be above the 10th percentile yet still pathologically growth restricted. A clear distinction between FGR and SGA would improve the precision of the review, particularly since this conflation is common in the broader literature.

We thank the reviewer for raising this concern, as we are aware that the terms SGA and FGR are often misused in the literature. We hope the addition of the following text (italicised) after the first mention of FGR in the introduction section distinguishes between SGA and FGR.

> **Line 50:** Disruption in substrate transfer can alter the trajectory of fetal growth, leading to babies being born with pathological fetal growth restriction (FGR) (Soothill *et al.*, 1987; Economides & Nicolaides, 1989; Nicolaides *et al.*, 1989; Economides *et al.*, 1991; Robinson, 1994; Owens, 1995). *Importantly, FGR is not synonymous with small for gestational age (SGA; <10th birthweight centile, where babies are genetically destined to be small but are otherwise healthy. In FGR, genetic growth potential is not met.*

Throughout the remainder of the review, we avoid using the term "SGA". We refer to fetuses as either FGR or non-FGR based solely on exposure to a model of a pathological condition (placental insufficiency, MNR or hypoxia) during pregnancy and on whether mean fetal weight compared to controls was reduced or not.

Conflation of nutrient restriction and hypoglycaemia

The focus on hypoglycemia and its cardiovascular consequences is appropriate and consistent with the stated aim of the review. However, many of the studies cited involve global maternal nutrient restriction, which cannot be equated with isolated fetal hypoglycemia. MNR typically results in multiple deficits such as amino acids and lipids, and attributing downstream cardiovascular effects solely to low glucose oversimplifies the biology. Greater emphasis on nutrient specific placental knockout models would strengthen this point. The graphical abstract and several figures for example Figures 1, 3, 4, 5, 6 should be revised to accurately reflect global nutrient deficiency rather than labelling these models as low glucose.

We thank the reviewer for raising this point and acknowledge that the primary focus on glucose may be viewed as a simplistic approach to understanding the mechanisms underlying cardiac programming, as the delivery of many other nutrients to the fetus is likely to be reduced as well. With that said, glucose is the primary fuel source for the developing fetal heart, with a switch to preferential use of lipids after birth. Furthermore, in most of the papers cited in our review, reduced fetal glucose concentration is among the most well-characterised and consistent substrate deficiency reported as a study outcome, alongside oxygen. Only a few studies measured other substrates in the fetal circulation.

We have first amended the abstract to remove the text that suggests hypoglycaemia "occurs in isolation" when describing other pregnancy complications other than placental insufficiency to avoid making bold statements.

Line 33: However, other common pregnancy complications exist, where fetal hypoxaemia or hypoglycaemia *may or may not occur.*

We hope that the following text in the introduction provides the reader with a rationale for why glucose is the primary substrate under focus.

> **Line 62:** *For instance, FGR can be due to factors of maternal, placental or fetal origin that reduce the capacity of the placenta to deliver* O_2 *and nutrients (primarily glucose, the major fuel source in fetal life), but also other nutrients such as amino acids and fatty acids (FAs)), termed placental insufficiency (PI), of which fetal hypoxaemia and hypoglycaemia are hallmarks (Soothill et al., 1987; Economides & Nicolaides, 1989; Nicolaides et al., 1989; Economides et al., 1991).*

We acknowledge that the machinery required for the transition to using FAs increase in late gestation; however, FAs do not cross the placenta as well as glucose, and it is not until the baby starts consuming milk rich in FAs that this is the preferential substrate for energy. In

fact, *Economides et al.,* describes that although some SGA human fetuses may have hypertriglyceridaemia and efficiently metabolise these FAs into ATP, this process requires oxygen, which is low the *in utero* compared to *ex utero* environment. Nonetheless, we have also amended Figure 1 to clearly state that nutrients (glucose) can cross the placenta and that this transfer can be reduced to the developing the fetus. The other figures mentioned by the reviewer (4, 5 and 6) have been removed from the manuscript.

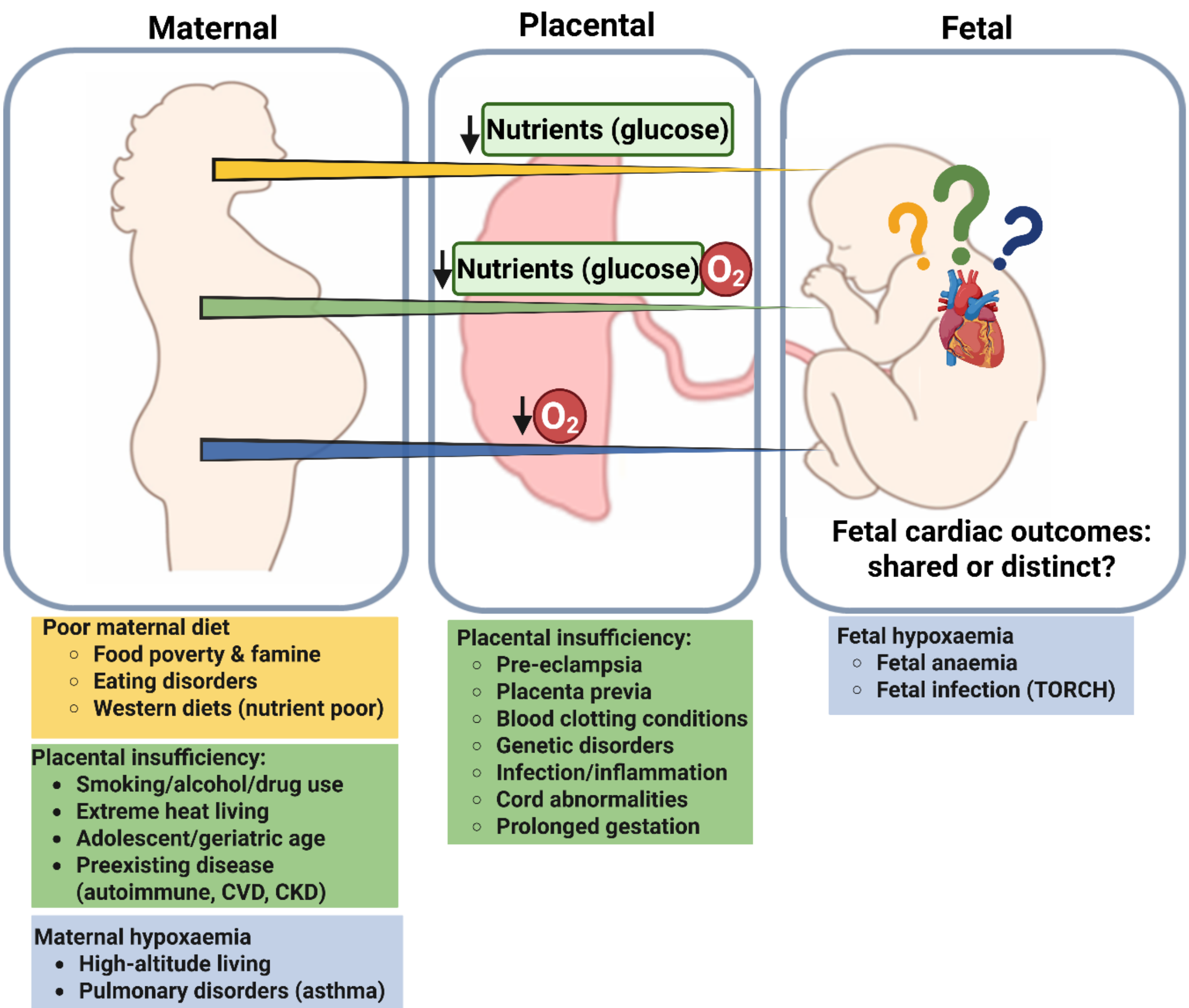

Revised Figure 1: Pregnancy complications leading to fetal hypoxaemia and/or hypoglycaemia and their impact on the developing fetal heart remain largely unknown. During pregnancy, oxygen (O_2) and nutrients, such as glucose, are transferred across the placenta from the maternal to the fetal unit. Many pregnancy complications of maternal, placental, or fetal origin can lead to reduced oxygen in the fetal circulation (blue), reduced nutrients such as glucose (yellow), or reduced oxygen and glucose (green) to the developing fetus. However, the independent and potentially synergistic roles of oxygen and glucose on fetal heart development due to poor maternal diet (yellow), placental insufficiency (green), and maternal hypoxaemia (blue) remain unclear. Cardiovascular disease (CVD), chronic kidney disease (CKD); TORCH (Toxoplasmosis, Other infections (syphilis, varicella, and parvovirus), Rubella, Cytomegalovirus, and Herpes simplex). This figure was made in Biorender.

We hope that the additional text (italicised) in the conclusion reiterates this fact to readers.

> **Line 779:** The interplay between these factors highlights the complexity of fetal cardiac programming. *In fact, the level of complexity extends beyond what this review sought to simplify, since we focused on glucose and oxygen, the main energy sources in fetal life and the most characterised nutrient deficiencies; however, other nutrients also play their own roles and they require further disentangling.*

Placental and oxygen transfer considerations

The authors acknowledge that early MNR can impair placental development and oxygen transfer, but this issue could be addressed more thoroughly. For example, comparisons with placental insufficiency induced IUGR PI IUGR, which involves reduced transfer of both oxygen and multiple nutrients, may provide useful context. Additionally, distinguishing between pre placental, placental, and post placental hypoxia see Kingdom and Kaufmann, 1997 PMID: 9364596 would add nuance, as these conditions produce distinct maternal and fetal phenotypes with potentially different cardiovascular outcomes.

We thank the reviewer for raising this point. Early-onset MNR during a period of rapid placental development can result in placental dysfunction. This contrasts with late onset MNR, which occurs after most placental growth has taken place and therefore has less impact on placental function and maintained oxygen delivery to the fetus. As such, we agree that it is important to highlight to the reader that the type of substrate deficiency caused by MNR depends on gestational timing, an important factor to consider when comparing fetal cardiac outcomes between early onset and late onset MNR.

Upon the first mention of MNR, the following text (italicised) has been added to highlight that timing of the insult matters.

> **Line 68:** For example, maternal nutrient restriction (MNR) reduces fetal nutrient delivery, resulting in fetal hypoglycaemia, *which, depending on the timing, severity and duration of the insult,* can leave an intact uteroplacental unit that maintains fetal O_2 delivery (Morrison, 2008) (Figure 1).

In the section describing animal models of MNR, the following text has been added (in italics).

> **Line 144:** A range of animal models have also been developed to recapitulate early and late gestation onset global restriction of maternal nutrients, which can result in reduced delivery of nutrients to the fetus, of which fetal hypoglycaemia is best characterised. *If onset is in late gestation, placental weight is maintained and there is no fetal hypoxaemia; however, early onset models can impair placental development, leading to reduced placental and fetal weight, like PI.*

In the MNR fetal cardiac outcome section, we have provided further mention of early onset MNR, which can result in reduced fetal body and/or heart weight, likely due to a degree of placental dysfunction.

> **Line 281:** However, in some instances, when MNR occurs early in gestation during rapid placental development, dysfunction of the placenta can arise, leading to phenotypes similar to PI-FGR, such as reduced fetal body and/or heart weight (Woodall *et al.*, 1996; Ozaki *et al.*, 2001; Edwards & McMillen, 2002; Vonnahme *et al.*, 2003; Camacho *et al.*, 2012; Elias *et al.*, 2016; Oliveira *et al.*, 2017; Nevin *et al.*, 2018; Pereira *et al.*, 2021).

We agree that this could be elaborated on further. However, given the reviewing editors comments on the already large word count and that most early onset MNR studies do not conduct functional or structural assessment of the placenta to confirm placental dysfunction, we hope that the additions listed above will suffice.

We also thank the reviewer for highlighting that not all types of fetal hypoxaemia are the same, and that the consequences for the fetal heart depend on the cause of hypoxaemia. The Kingdom and Kaufman reference provided effectively addresses this point. Ideally, we would like to do a more in depth comparison with how each of these conditions may influence cardiovascular outcomes; however, in light of the already large word count and requirement to cut text, we have added extra detail at the start of the hypoxia section to highlight the importance of distinguishing between pre-placental hypoxia, which may arise from low oxygen levels in the mother due to anaemia or high-altitude living, placental hypoxia resulting from poor uterine blood flow, and post-placental hypoxia resulting in poor transfer of oxygen to the fetus with the Kingdom and Kaufman reference added to support this statement.

Line 390: *Indeed, differences in fetal outcomes depend on the origin of hypoxia, which can arise pre-placentally (MH) or post-placentally (fetal anaemia) impacting how the fetus responds (Kingdom & Kaufmann, 1997).*

Minor comments:

Figure 1: The colour coded bubbles on the fetal heart are confusing and should be clarified with a legend or key, similar to the explanatory boxes below.

We thank the reviewer for the feedback regarding improving the clarity of the symbols. Our intent was to conceptualise the lack of clarity in how deficiency of different substrates may lead to different fetal cardiac outcomes, which this review aimed to address. Please refer to revised Figure 1 above (page 4) that has been simplified and a clear question posed: “Fetal cardiac outcomes: shared or distinct?”. In addition, the legend of Figure 1 has been expanded to explain the pregnancy complication that the colours represent.

Figure 1 legend: However, the independent and potentially synergistic roles of oxygen and glucose due to poor maternal diet (yellow), placental insufficiency (green), and hypoxaemia (blue) on fetal heart development remain unclear.

Figures 3, 4, 5, 6: As noted above, these should not depict MNR effects solely as low glucose. Depending on the model, animals may fall under the combined oxygen and glucose deprivation category.

We have removed Figures 4, 5, and 6 from the review upon revising the manuscript in response to reviewer #2's feedback. We have also amended the graphical abstract to state "low nutrients, e.g., glucose," at the top of the image to avoid depicting MNR as solely glucose from the beginning of the review. We wish to keep the text "low glucose" in Figure 3, as this figure is reporting outcomes measured within specific studies that for the most part did not measure other nutrients in the fetal circulation.

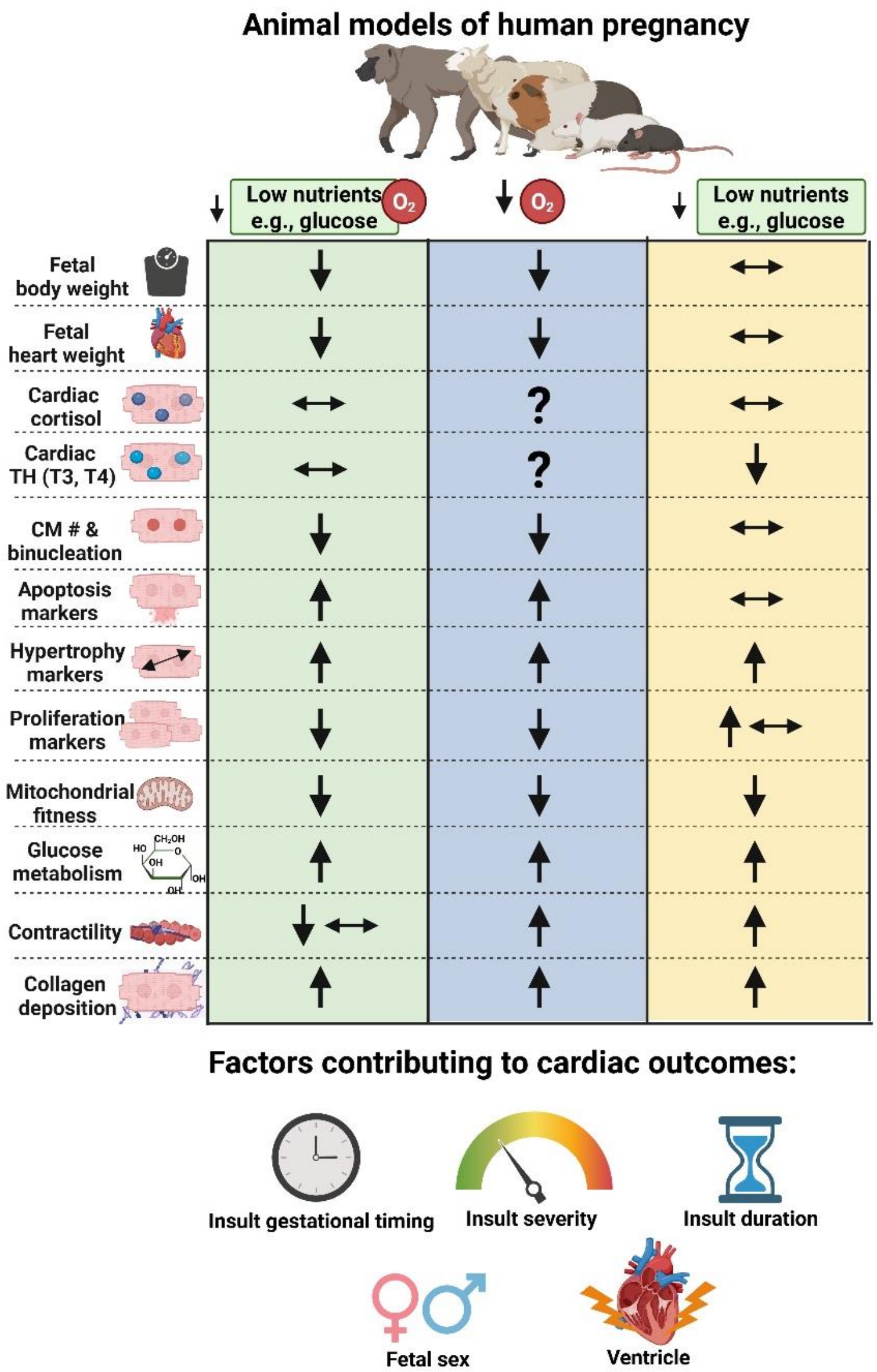

Revised graphical abstract

Figures 8 and 9: While interesting, these figures do not directly align with the stated focus on glucose versus oxygen deprivation, as they address more general issues of sex differences and therapeutic approaches in FGR. The authors may wish to clarify their relevance or adjust emphasis.

Thank you for finding the Figures interesting. We have decided to retain Figure 8 as fetal sex is a critical factor in cardiac programming and is often overlooked as a variable in medical research. Thus, in addition to the focus on oxygen and glucose, we addressed their role in parallel with sex hormones, because disentangling the effects of oxygen and glucose on the fetal heart requires consideration of whether the fetus is male or female. We believe we can provide the reader with a stronger rationale, so the following sentence has been added at the start of the fetal sex section.

> **Line 544:** To comprehensively understand how deficits in oxygen and nutrients like glucose impact fetal heart development, it is important to consider the sex of the fetus under investigation.

Moreover, we hope that the changes made to the graphical abstract (see page 7 of this document), which now displays fetal sex and ventricles as contributing factors to consider when understanding how pregnancy complications can lead to cardiac risk profiles, better integrates sex into the review.

Given concerns about the length of the review and as suggested by reviewer #2, we have decided to omit the section on therapeutic approaches in the FGR from this review to maintain focus on the topic and reduce word count. Thus, the associated Figure 9 has been cut from the review.

Line 107: Specify "non human primates" rather than simply "primates."

We thank the reviewer for raising this point, as we were not referring to humans and meant non-human primates. This change has been made on Line 107.

Line 417 to 420: Expand on the distinction between different forms of hypoxia pre, placental, post placental. See Kingdom and Kaufmann, 1997 PMID: 9364596. This is important as the consequences to mom and baby differ based on which compartments the hypoxia is impacting.

We agree that it is important to highlight to readers that high-risk fetal cardiac phenotypes may depend on which compartment the hypoxia compromise occurs. Thank you for raising this. We have added the following text:

> **Line 390:** *Indeed, differences in fetal outcomes depend on the origin of hypoxia, which can arise pre-placentally (MH) or post-placentally (fetal anaemia) dictating how the fetus responds (Kingdom & Kaufmann, 1997).*

Summary:

This review is thorough, well written, and addresses an important topic with strong potential impact. However, improved precision in terminology and interpretation particularly regarding distinctions between hypoglycemia, MNR, and FGR versus SGA will be critical to ensuring clarity and accuracy. Addressing these issues will significantly enhance the strength and reliability of the manuscript.

We appreciate the reviewers positive feedback and for taking the time to review this manuscript carefully. We hope that the amendments made based on comments provided have improved the quality of our manuscript.

Referee #2:

This comprehensive review by Bertossa et al. provides a detailed exploration of how placental insufficiency, maternal nutrient restriction (MNR), and fetal hypoxemia individually and collectively influence the fetal origins of cardiovascular disease. A key strength of the manuscript is its thorough analysis of cardiac programming across multiple domains, including growth, hormonal regulation, metabolism, contractility, and cardiac structure. The review offers a unique contribution by integrating an in-depth discussion of various animal models and the impact of developmental timing on cardiac outcomes. The paper also effectively addresses sexual dimorphism and ventricle-specific responses, highlighting differential right vs. left ventricular responses under conditions of nutrient and/or oxygen restriction. Despite substantial content and valuable insight, several areas require attention.

Primary concerns:

Writing is overly dense and could benefit from more selective focus and a clearer synthesis of key messages. For example, there is redundancy and repetition that cause the reader to lose essential points being made by the authors. One instance would be the discussion of mitochondrial dysfunction in all three conditions (PI, MNR and hypoxemia) being discussed in a nearly identical manner (lines 235-252, 368-378, 460-470); it seems this could be better integrated to reduce repetition and improve flow. Similar issues are present for several other concepts and require attention to enhance clarity and impact.

We thank the reviewer for raising this. Our intention was to ensure that each section could stand alone. Upon reflection, this can make the text repetitive and dense at times. Any overlapping descriptions have been condensed to streamline the information. The reviewer highlights the mitochondrial dysfunction section, and we have cut/condensed this (lines 241-244, 248-251, 371-372-375-377, 379-382, 470-473, 475-477, 628-640). Total words cut ~250 words.

The reviewer also highlighted several other concepts that require attention. We believe that the concepts that require condensing are those related to growth profiles (lines 314-315, 318-

320, 434-436), contractility (390, 395, 521-523, 529-531) and hormonal regulation (349, 353, 366-367) and have revised accordingly.

Figures. While the figures are informative, these (particularly Figures 4-6) are overcrowded and could be streamlined to distill the complex information into a more easily digestible form. I realize that the amount of information being visualized is quite large, but I did not find the current presentation in these 3 figures in particular to be effective.

We appreciate the reviewers feedback on these Figures whilst acknowledging the complexity of information that we are trying to represent. We agree that there is an opportunity to streamline this information from all 3 conditions to allow the reader to compare to fetal cardiac outcomes in a more digestible way. We believe the graphical abstract in part achieves this by comparing the cardiac outcomes from placental insufficiency, MNR and hypoxia and have cut Figures 4-6 from the review. Cutting these Figures and their associated legends has further reduced the manuscripts word count by ~300 words.

The review includes what may be viewed as an excessive proportion of self-citations, which raises concerns about selection bias and whether the review adequately represents the broader literature. Please ensure that all references are necessary (self-citations and otherwise).

We agree that self-citing is a common problem in academia and a valid concern to raise, particularly when a given reference is not relevant to the topic at hand. This is a highly collaborative field with nine co-authors on this paper being first and/or co-authors on considerable bodies of work directly relevant to this field.

Overall, we believe that the original 440 references underscore the attempt made to cover the broader literature. We acknowledge the reviewer's concern that 440 references in themselves are significant, and we have gone back through the manuscript to remove unnecessary references where appropriate, further reducing the total word count. In instances where there are more than 6-8 references supporting a statement, we have reduced this to 3-5 and kept only the seminal or most recent confirmatory paper for that statement. In many cases, the multiple citations reflect that similar outcomes were found in different models, and this is important in assessing the likelihood of a change being associated with low oxygen and/or glucose and thus also occur in humans.

Thus, a total of 120 citations have been removed from the manuscript. Of the remaining 319 references, most co-authors have a self-citation rate of ~2-3%, with the highest at 9.7%. We sought to minimise self-citation, as recommended by the Journal of Physiology guidelines.

Limited critical analysis. The review does a commendable job of summarizing existing literature but lacks a sufficient critical evaluation or discussion of contradictory findings; this somewhat restricts its utility as an objective evidence base for the field. Just one example, lines 277-283: "Some fetuses remain normotensive" despite PI, but then states "However, these fetuses may be maintaining blood pressure differently" with no follow-up for why this may be the case; no discussion of why 30-50% of studies show no HTN effect or methodological differences that might explain discrepancies. Animal model limitations are noted but not followed by how these limitations affect conclusions or translational relevance; this should be evaluated more fully given the focus of the review on animal models. Essentially, there are many missed opportunities to take the nice synthesis of existing literature a step further by reconciling conflicting or mixed findings.

We thank the reviewer for raising this, whilst acknowledging our attempt to summarise existing literature. We found this to be difficult as studies used different species, models of FGR and reported different outcomes. With that being said, we agree that there are parts of the review where differences in the literature could be expanded upon. We have edited the paper to take advantage of these opportunities whilst remaining mindful of the word count.

In the MNR section, we make direct comparisons to reported PI outcomes related to growth, hormonal, metabolic and contractility:

> **Lines**: 273, 275, 287, 291, 293, 306, 315, 328, 330, 334, 337, 359, 362, 366

In the hypoxia section, we make direct comparison to reported outcomes in PI and MNR:

> **Lines**: 388. 403, 407, 413, 425, 437, 454, 479, 498

The following text has been added to the end of the conclusion as a last concluding remark to remind the readers of the many factors at play that can influence the cardiac outcomes described.

> **Lines 783:** *Nonetheless, drawing conclusions on cardiac outcomes across studies proved to be difficult, as outcomes are influenced by not only substrates, sex and ventricles examined, but also other experimental design factors including species and litter size, model of FGR used and outcome measures. Despite these complexities, this review highlights promising mechanistic pathways and potential candidate targets for intervention that could protect against the fetal origins of CVD.*

Translation to humans: A more detailed discussion of risks/limitations/challenges to the translation of findings to humans is required.

We agree that this is an important point. In the first mention of the necessary use of animal models to recapitulate human pregnancy in the section titled *Animal models that have similar placental and fetal heart development to humans,* we state on line 220 "Animal models can be used to recapitulate human pregnancy, including non-human primates (such as macaques and baboons), sheep, and rodents (including guinea pigs, mice, and rats), each with reproductive similarities and differences to humans, which must be carefully considered when translating relevant findings into the human context." Specific examples of

reproductive similarities and differences follow this. However, we hope that the addition of text that directly follows this, as a concluding statement in this section, reminds the reader that this reproductive variability presents challenges in translating findings to humans.

> **Line 125:** While animal models are useful, reproductive variability between species can introduce challenges when comparing study outcomes and translating findings into the human context.

Minor: Inconsistent abbreviation use. Sometimes cardiomyocyte is spelled out, other places it is abbreviated as CM; same with FGR (sometimes "growth-restricted") and MNR (sometimes "nutrient restriction"). Consider using fewer abbreviations and/or providing an abbreviations table for the manuscript.
We thank the reviewer for raising these inconsistencies. We agree that an abbreviation table would be helpful for the reader; however, the Journal of Physiology does not allow abbreviation tables and may not be best suited, given the length of the manuscript as it stands. Instead, we have thoroughly revised the manuscript, and in the first mention of common abbreviations (i.e., CM, FGR, MNR), they have been spelled out. We have avoided abbreviating words that are only used a few times.

Overall, this is a well-researched and ambitious review that addresses a critical topic in the developmental programming of cardiovascular disease. Revisions are necessary to improve its clarity and enhance critical analysis. I encourage the authors to refine and condense the manuscript to maximize the impact and accessibility of the important points and information they want to convey.
Thank you for taking the time to review this manuscript. We have carefully condensed the manuscript by reducing its length by ~3,000 words to improve clarity and impact.

REFERENCES

Camacho LE, Meyer AM, Neville TL, Hammer CJ, Redmer DA, Reynolds LP, Caton JS & Vonnahme KA. (2012). Neonatal hormone changes and growth in lambs born to dams receiving differing nutritional intakes and selenium supplementation during gestation. *Reproduction* **144,** 23-35.

Economides DL & Nicolaides KH. (1989). Blood glucose and oxygen tension levels in small-for-gestational-age fetuses. *American Journal of Obstetrics and Gynecology* **160,** 385-389.

Economides DL, Nicolaides KH & Campbell S. (1991). Metabolic and endocrine findings in appropriate and small for gestational age fetuses. *J Perinat Med* **19,** 97-105.

Edwards LJ & McMillen IC. (2002). Periconceptional nutrition programs development of the cardiovascular system in the fetal sheep. *Am J Physiol Regul Integr Comp Physiol* **283,** R669-679.

Elias AA, Ghaly A, Matushewski B, Regnault TR & Richardson BS. (2016). Maternal Nutrient Restriction in Guinea Pigs as an Animal Model for Inducing Fetal Growth Restriction. *Reprod Sci* **23,** 219-227.

Kingdom JC & Kaufmann P. (1997). Oxygen and placental villous development: origins of fetal hypoxia. *Placenta* **18,** 613-621; discussion 623-616.

Morrison JL. (2008). Sheep models of intrauterine growth restriction: fetal adaptations and consequences. *Clin Exp Pharmacol Physiol* **35,** 730-743.

Nevin CL, Formosa E, Maki Y, Matushewski B, Regnault TRH & Richardson BS. (2018). Maternal nutrient restriction in guinea pigs as an animal model for studying growth-restricted offspring with postnatal catch-up growth. *Am J Physiol Regul Integr Comp Physiol* **314,** R647-r654.

Nicolaides KH, Economides DL & Soothill PW. (1989). Blood gases, pH, and lactate in appropriate- and small-for-gestational-age fetuses. *Am J Obstet Gynecol* **161,** 996-1001.

Oliveira V, Silva Junior SD, de Carvalho MHC, Akamine EH, Michelini LC & Franco MC. (2017). Intrauterine growth restriction increases circulating mitochondrial DNA and Toll-like receptor 9 expression in adult offspring: could aerobic training counteract these adaptations? *Journal of Developmental Origins of Health and Disease* **8,** 236-243.

Owens J, Owens, PC, Robinson, JS. (1995). Experimental restriction of fetal growth. *Fetus and Neonate, Physiology and Clinical Applications***,** 139-175.

Ozaki T, Nishina H, Hanson MA & Poston L. (2001). Dietary restriction in pregnant rats causes gender-related hypertension and vascular dysfunction in offspring. *J Physiol* **530,** 141-152.

Pereira SP, Tavares LC, Duarte AI, Baldeiras I, Cunha-Oliveira T, Martins JD, Santos MS, Maloyan A, Moreno AJ, Cox LA, Li C, Nathanielsz PW, Nijland MJ & Oliveira PJ. (2021). Sex-dependent vulnerability of fetal nonhuman primate cardiac mitochondria to moderate maternal nutrient reduction. *Clin Sci (Lond)* **135,** 1103-1126.

Robinson J, Owens, JA and Owens, PC. (1994). Fetal growth and fetal growth retardation. *Textbook of Fetal Physiology***,** 83–94.

Soothill PW, Nicolaides KH & Campbell S. (1987). Prenatal asphyxia, hyperlacticaemia, hypoglycaemia, and erythroblastosis in growth retarded fetuses. *Br Med J (Clin Res Ed)* **294,** 1051-1053.

Vonnahme KA, Hess BW, Hansen TR, McCormick RJ, Rule DC, Moss GE, Murdoch WJ, Nijland MJ, Skinner DC, Nathanielsz PW & Ford SP. (2003). Maternal undernutrition from early- to mid-gestation leads to growth retardation, cardiac ventricular hypertrophy, and increased liver weight in the fetal sheep. *Biol Reprod* **69,** 133-140.

Woodall SM, Johnston BM, Breier BH & Gluckman PD. (1996). Chronic Maternal Undernutrition in the Rat Leads to Delayed Postnatal Growth and Elevated Blood Pressure of Offspring. *Pediatric Research* **40,** 438-443.

Dear Janna,

Re: JP-TR-2026-289670R1 "**Insights from animal models: Dissecting the independent roles of oxygen and nutrients in the fetal origins of cardiovascular disease** " by Melanie Rose Bertossa, Jack RT Darby, Ashley Meakin, Mitchell C Lock, Steven KS Cho, Christopher K Macgowan, Mike Seed, Michael D Wiese, and Janna L Morrison

We are pleased to tell you that your paper has been accepted for publication in The Journal of Physiology.

TRANSPARENT PEER REVIEW POLICY: To improve the transparency of its peer review process, The Journal of Physiology publishes online as supporting information the peer review history of all articles accepted for publication. Readers will have access to decision letters, including Editors' comments and referee reports, for each version of the manuscript, as well as any author responses to peer review comments. Referees can decide whether or not they wish to be named on the peer review history document.

The last Word (or similar) version of the manuscript provided will be used by the Production Editor to prepare your proof. When this is ready you will receive an email containing a link to Wiley's Online Proofing System. The proof should be thoroughly checked and corrected as promptly as possible.

Authors should note that it is too late at this point to offer corrections prior to proofing. Major corrections at proof stage, such as changes to figures, will be referred to the Editors for approval before they can be incorporated. Only minor changes, such as to style and consistency, should be made at proof stage. Changes that need to be made after proof stage will usually require a formal correction notice.

All queries at proof stage should be sent to: TJP@wiley.com.

Are you on Twitter? Once your paper is online, why not share your achievement with your followers? Please tag The Journal (@jphysiol) in any tweets and we will share your accepted paper with our 30,000 followers!

Best wishes,

Laura Bennet
Senior Editor
The Journal of Physiology

P.S. - You can help your research get the attention it deserves! Check out Wiley's free Promotion Guide for best-practice recommendations for promoting your work at www.wileyauthors.com/eeo/guide. You can learn more about Wiley Editing Services which offers professional video, design, and writing services to create shareable video abstracts, infographics, conference posters, lay summaries, and research news stories for your research at www.wileyauthors.com/eeo/promotion.

• IMPORTANT NOTICE ABOUT OPEN ACCESS: To assist authors whose funding agencies mandate immediate public access to published research findings, The Journal of Physiology allows authors to pay an Open Access (OA) fee to have their papers made freely available immediately on publication.

The Corresponding Author will receive an email from Wiley with details on how to register or log-in to Wiley Authors Services where you will be able to place an order.

You can check if your funder or institution has a Wiley Open Access Account here: https://authorservices.wiley.com/author-resources/Journal-Authors/licensing-and-open-access/open-access/author-compliance-tool.html.

EDITOR COMMENTS

Reviewing Editor:

Thank you for the significant efforts to address the reviewers concerns and reduce the word count. The next version has been substantially improved. Thank you for your excellent contribution to the field and The Journal.

REFEREE COMMENTS

Referee #1:

The authors have sufficiently addressed my concerns, providing clarity in previous areas of ambiguity.

Referee #2:

Thank you for thoughtfully addressing my comments and suggestions.
